# Can explainable AI scaffolding reduce cognitive load and enhance embodied expression? A quasi-experimental study of multilingual vocal learning in Bel Canto education

**DOI:** 10.3389/fpsyg.2026.1835027

**Published:** 2026-06-23

**Authors:** Yuyi Sun, Xiaodi Wang, Xuefeng Wei

**Affiliations:** 1 College of Education, Ludong University, Yantai, Shandong, China; 2 Institute for Education and Treatment of Problematic Youth, Ludong University, Yantai, China

**Keywords:** Bel Canto education, cognitive load, embodied expression, explainable artificial intelligence, scaffolding learning

## Abstract

Exploring how artificial intelligence technology can effectively support music education has become a critical issue in the digital era. This study addresses the high cognitive load and embodied expression difficulties faced by bel canto students when learning multilingual vocal works, designing and validating the educational effectiveness of an explainable AI scaffolding learning system. The study employed a 2 × 3 mixed-factor quasi-experimental design, with 64 college students from the Conservatory of Music allocated to the XAI scaffolding group and the traditional AI-assisted group through stratified assignment for a 16-week intervention on the learning of vocal music works in Italian, German, and French. Data collection integrated multimodal measurements including cognitive load scales, expert ratings, motion capture, and eye-tracking, with analysis using repeated-measures mixed-factor ANOVA, Bootstrap-based mediation analysis, and thematic analysis. The study found that the XAI scaffolding group's cognitive load reduction reached 38.9%, significantly higher than the control group's 17.0% (*p* < 0.001, *d* = 2.36, 95% CI [1.72, 2.99]), with extraneous cognitive load reduction of 41.2% being the primary contribution; embodied expression total scores improved by 45.0%, with body posture, facial expression, and gesture use dimensions all significantly superior to the control group; language complexity moderated XAI effects, with the advantage most pronounced in German learning (*g* = 2.71); the three sequence-disambiguation analyses converged on an independent contribution of language complexity beyond cumulative learning, with the language × group interaction remaining significant after cumulative-exposure adjustment ($\eta_{p}^{2}$ = 0.078, *p* = 0.007), although complete causal separation awaits future counterbalanced replication; cognitive load played a partial mediating role between XAI scaffolding and embodied expression, with indirect effects accounting for 36.6% of total effects; metacognitive level significantly moderated the mediation pathway; qualitative analysis revealed an “understanding-experiencing-integrating” learning pathway mechanism. This study provides empirical evidence of the potential value of explainable AI in vocal education, offering preliminary theoretical insights and practical guidance for transparent design of intelligent music education systems. These findings warrant replication with larger, multi-site samples and counterbalanced sequencing designs before direct generalization to educational practice.

## 1 Introduction

Digital transformation is profoundly reshaping the teaching models and learning experiences of music education, with the application of artificial intelligence technology in vocal training increasingly widespread, injecting new vitality into traditional bel canto teaching (Li et al., 2025; Song, 2025). Students majoring in bel canto face complex cognitive challenges when learning vocal music works in multiple languages such as Italian, German, and French. They need to simultaneously process multidimensional information such as phonetic pronunciation, musical melody, and emotional expression. According to cognitive load theory (Sweller, 1988), this high-intensity information processing process significantly increases cognitive load, particularly when learners must coordinate auditory, motor, and linguistic subsystems concurrently (Song, 2025). Although current AI-assisted vocal learning systems can provide real-time feedback and personalized guidance, their decision-making processes lack transparency, making it difficult for students to understand why the system provides specific technical suggestions or evaluation results (Khosravi et al., 2022). This “black box” characteristic severely restricts the deep application and value realization of AI technology in vocal education (Conati et al., 2021; Türkmen, 2025). The introduction of explainable AI technology provides new insights for resolving this dilemma. By making algorithmic decision-making processes transparent and understandable, it can enhance students' trust in AI systems, promote their active acceptance and utilization of learning scaffolds provided by the system, thereby optimizing cognitive resource allocation and improving learning effectiveness and embodied expression ability in multilingual vocal works (Farrow, 2023).

Research on AI technology applications in music education has made substantial progress in recent years. AI-driven tools have been applied across various musical domains, including rhythm learning (Koong et al., 2025), automated performance assessment, and real-time visual feedback in instrumental and vocal pedagogy (Lã and Fiuza, 2022). In the context of general music learning, Song's (2025) structural equation modeling research revealed the mediating role of technology acceptance in AI-assisted music learning, with students' perceived usefulness and ease of use directly affecting cognitive load management effectiveness. Li et al.'s (2025) mixed-method study showed that AI chatbot feedback combined with self-assessment tools can significantly improve vocal learners' metacognitive abilities. However, the majority of existing AI applications in music education have been designed for instrumental or general music contexts, with comparatively limited attention to the unique demands of vocal music learning. Regarding theoretical frameworks for explainable AI in education, Khosravi et al. (2022) proposed the XAI-ED framework providing guidance across six key dimensions for developing educational AI tools, while Conati et al.'s (2021) empirical research further demonstrated that individual characteristics moderate explanation effectiveness. Koong et al. (2025) demonstrated through quasi-experimental design that mobile technology-integrated dynamic assessment systems can significantly improve students' music rhythm learning achievement without increasing cognitive load. In embodied cognition and vocal performance research, Juntunen et al. (2023) argued from a phenomenological perspective for the embodied nature of singing expression, and Forbes et al. (2024) revealed embodied characteristics of vocal teachers' musical identity through interpretive phenomenological analysis. Research on visual feedback technology applications in vocal pedagogy shows that real-time visual feedback has become a key teaching method in current and future vocal classrooms (Lã and Fiuza, 2022). However, comprehensive literature analysis reveals obvious gaps in research specifically targeting vocal training with explainable AI. Unlike instrumental performance, singing involves the body itself as the instrument, posing unique challenges for embodied learning that existing AI research fails to adequately address (Forbes et al., 2024). Existing research fails to adequately consider the embodied, aesthetic, and highly personalized characteristics of vocal learning.

This study focuses on designing and empirically validating an explainable AI scaffolding learning system, constructing an XAI framework suitable for multilingual vocal learning in bel canto majors by integrating cognitive load theory (Sweller, 1988; Sweller et al., 2019), embodied cognition theory (Reybrouck and Schiavio, 2024), and scaffolding learning theory (Wood et al., 1976; Vygotsky, 1978). Innovation is manifested in three dimensions: theoretical innovation involves introducing explainable AI into vocal education, expanding the application boundaries of cognitive load theory in music learning, and establishing domain-specific design principles for XAI educational applications; technical innovation develops a vocal learning system integrating SHAP and LIME technologies, achieving multi-level explanation of AI decision-making processes, including global explanation, local explanation, and counterfactual explanation, enabling students to understand how the system evaluates their pronunciation accuracy, timbre quality, and emotional expression; methodological innovation employs a 16-week longitudinal quasi-experimental design, using multimodal data collection (cognitive load scales, expert ratings, eye-tracking, motion capture) and mixed-methods analysis to analyze dynamic change trajectories of cognitive load and comprehensively evaluate embodied expression improvement pathways. The methodological design further incorporates three pre-planned strategies to strengthen causal inference within the practical constraints of a conservatory teaching setting. A multi-layered blinding architecture combines rater blinding (expert evaluators were blind to group assignment), interviewer blinding (research assistants conducting qualitative interviews were uninformed of participants' conditions), and participant condition masking supported by a manipulation-check procedure at Weeks 8 and 16 to verify that learners could not reliably identify their assigned system type. A sequence-disambiguation analytical plan was prespecified to separate language complexity from cumulative learning effects, including segmented within-language slope comparison, cross-language first-week contrasts, and covariate-adjusted modeling that treats cumulative XAI exposure time as a continuous control. A suite of small-sample robustness analyses—Bootstrap resampling with bias-corrected confidence intervals, leave-one-out cross-validation, and sensitivity tests after outlier trimming and covariate adjustment—was prespecified to evaluate the stability of primary findings under the conditions characteristic of educational intervention research. The research will provide empirical evidence for intelligent teaching reform in music conservatories, promote responsible application of AI technology in vocal education, and ultimately serve the goal of cultivating high-level vocal talents with international vision and cross-cultural communication abilities.

## 2 Literature and theory

### 2.1 Applications of explainable AI in education

The application of explainable artificial intelligence technology in education is experiencing a critical transition from theoretical exploration to practical validation. The XAI-ED framework constructed by Khosravi et al. (2022) provides systematic guidance for developing educational AI tools, covering six core dimensions including stakeholders, explanation presentation methods, AI model categories, and human-centered design considerations. Explainability needs in educational environments differ essentially from general AI applications; transparency in the learning process concerns not only technical accuracy but also deeper issues such as learner autonomy, teacher-student interaction quality, and educational equity. Farrow's (2023) socio-technical perspective analysis reveals that explanation comprehensibility must be negotiated across different stakeholder groups; students need to understand why they receive specific feedback, teachers need to know how to supplement AI-provided support, and administrators focus on the rationality and fairness of system decisions.

Recent empirical research has begun validating XAI's actual impact on learning outcomes. Conijn et al.'s (2023) research on automated essay scoring systems found that students prefer to receive explanations about higher-level features (content, structure, coherence) rather than surface feature feedback, and this transparency significantly affects students' trust and learning motivation. Türkmen's (2025) systematic review analyzed 35 XAI educational application studies from 2019 to 2024, finding that while technical methods like SHAP and LIME can explain AI decisions, a huge gap remains in converting technical explainability into pedagogically meaningful insights. This conversion difficulty is particularly pronounced in skill-based learning such as music education, where learners need to understand not just scoring results but improvement pathways and practice strategies.

It is important to note that XAI explanations can be characterized along two complementary dimensions: explanation scope and explanation format. In terms of scope, explanations range from global explanations (revealing overall model behavior and feature importance across predictions), to local explanations (clarifying why a specific prediction was made for a particular input), to counterfactual explanations (indicating what minimal changes would alter the prediction outcome) (Khosravi et al., 2022; Conati et al., 2021). In terms of presentation format, the same explanation scope can be delivered through different modalities, including rule-based formats (presenting explicit if-then rules or threshold criteria), example-based formats (providing concrete instances for comparison), or mixed formats combining both approaches (Türkmen, 2025). In educational contexts, learners' preferences for explanation format may evolve with learning progress and task complexity, a dynamic that has received insufficient empirical attention in prior research. The present study examines both dimensions, implementing global, local, and counterfactual explanations at the system design level, while tracking learners' actual engagement with rule-based and example-based presentation formats during the learning process.

### 2.2 Development trajectory of scaffolding learning theory

Scaffolding learning theory achieved a paradigm shift from static presetting to dynamic adaptation during 2022–2024, particularly evident in technology-enhanced learning environments. Faber et al.'s (2024) randomized controlled trial demonstrated that adaptive scaffolding in gamified learning can reduce medical students' cognitive load while improving operational speed, showing significant advantages over fixed scaffolding. Core mechanisms of scaffolding effectiveness include first-order domain-specific indicators such as accuracy, systematicity, and thoroughness, as well as second-order metacognitive indicators including monitoring trajectories and self-regulated learning scores Faber et al., (2023). Munshi et al.'s (2023) research on students in adaptive learning environments further refined these findings, showing strategic hints are more effective than encouragement hints, and scaffolding effects differ significantly between high and low-level learners.

The theoretical foundation of dynamic scaffolding systems is expanding from Vygotsky's zone of proximal development concept to emerging fields such as computational thinking scaffolding and generative AI adaptive support. Shao et al.'s (2023) meta-analysis examined multiple types of regulated learning scaffolding including scripts, group awareness tools, and intelligent tutoring agents, finding that scaffolding effectiveness depends simultaneously on macro design features and micro implementation details. This multi-level perspective is crucial for understanding scaffolding mechanisms in complex learning environments. Wang et al.'s (2023) research demonstrated how computer-based scaffolds differentially affect metacognitive monitoring accuracy and problem-solving efficiency, providing empirical evidence for designing personalized scaffolding strategies.

### 2.3 Applications of cognitive load theory in music learning

Cognitive load theory, originally proposed by Sweller (1988), provides an important framework for understanding the complex cognitive processes of learning. The theory was further refined through the distinction of three cognitive load components: intrinsic load, extraneous load, and germane load (Sweller et al., 2019). Intrinsic cognitive load arises from the inherent complexity of the learning material and the element interactivity it demands, which cannot be directly altered by instructional design. Extraneous cognitive load is generated by suboptimal instructional design or presentation, consuming cognitive resources without contributing to learning; reducing extraneous load is a primary objective of effective instructional interventions. Germane cognitive load refers to the cognitive effort devoted to schema construction and automation, representing productive engagement with the learning material. In music learning, these three components have distinct manifestations: intrinsic load corresponds to the inherent complexity of musical tasks (e.g., the element interactivity between pitch, rhythm, lyrics, and expression), extraneous load may be induced by poorly designed feedback interfaces or unclear instructional guidance, and germane load reflects the learner's active effort to build integrated performance schemas. Measuring these components separately is critical for understanding how instructional interventions operate; Leppink et al. (2013) developed a validated instrument that differentiates the three load types, providing a methodological basis for component-level analysis in educational research.

In the specific context of vocal music learning, cognitive load exhibits unique multidimensional characteristics, involving coordinated work of multiple cognitive subsystems including auditory processing, motor coordination, and emotional expression. When bel canto students learn multilingual works, intrinsic load is elevated by the simultaneous demands of phonetic accuracy, melodic production, and interpretive expression across unfamiliar linguistic systems. Extraneous load may increase when AI feedback systems provide opaque or difficult-to-interpret suggestions, forcing learners to expend cognitive resources on deciphering system outputs rather than improving performance. Germane load, by contrast, can be fostered when feedback is transparent and learners can direct cognitive effort toward building integrated phonetic-musical-expressive schemas. Recent research has further illuminated the key role of individual differences in cognitive load management. Que et al.'s (2023) research using eye-tracking technology to study the impact of background music on reading tasks found that high-level learners and students accustomed to using background music experience lower cognitive load, providing important insights for designing personalized music learning scaffolds. Zhang et al.'s (2020) research used gamification methods to support elementary students' fraction concept learning, effectively managing cognitive load and improving learning outcomes through carefully designed digital games.

Song's (2025) research used structural equation modeling to analyze the impact of AI-assisted tools on music education students' cognitive load, finding technology acceptance plays a key mediating role in cognitive load management. Drai-Zerbib and Perruchet's (2024) experimental research found that musicians significantly outperform non-musicians in spatial working memory tasks [*F*_(1, 56)_ = 7.52, *p* = 0.008, $\eta_{p}^{2}$ = 0.118] and demonstrate higher metacognitive accuracy, with 46.34% of musicians able to accurately assess their own performance compared to only 14.63% of non-musicians. This metacognitive advantage has important significance for self-regulated learning and gradual scaffold withdrawal. Taken together, the three-component framework of cognitive load theory suggests that effective interventions in vocal music learning should aim to reduce extraneous load (e.g., through transparent AI feedback), maintain manageable intrinsic load (through appropriate task sequencing), and promote germane load (by facilitating schema construction). This theoretical lens directly informs the design rationale and evaluation framework of the present study.

### 2.4 Embodied cognition and musical expressiveness

Embodied cognition theory provides a new perspective for understanding the mind-body integration process in vocal learning, emphasizing the core position of bodily experience in musical understanding and expression. Alves and Nogueira's (2024) qualitative research on three clarinet expert teachers proposed “embodied consciousness” as a new competency domain in music performance pedagogy, with expert teachers evoking learners' embodied memories and sensations through metaphorical language and bodily gestures, activating kinesthetic, sensory, episodic, semantic, and emotional memory patterns. The embodied characteristics of vocal learning are even more pronounced, as voice originates directly from the body, and singing self is difficult to separate from individual self. This expressive mode without external instrumental mediation requires scaffold design to consider sensorimotor dimensions (Forbes et al., 2024).

Sensorimotor synchronization plays a key role in music learning. Carrer et al.'s (2023) research on 305 school-age children revealed developmental trajectories of rhythmic synchronization and body coordination abilities, providing basis for designing embodied scaffolds adapted to developmental stages. aarikallio et al.'s (2023) systematic review analyzed the effects of Dalcroze methods and music-movement integration interventions on vulnerable populations, demonstrating that body-based scaffolds provide alternative pathways for skill development, with this multimodal approach particularly effective for learners who struggle with purely cognitive or auditory teaching. The embodied cognition perspective on music learning emphasizes the tight integration of perception, action, and cognition, challenging the traditional “sandwich” cognitive model and providing theoretical foundation for designing scaffold systems more aligned with music learning's nature (Reybrouck and Schiavio, 2024).

### 2.5 Theoretical integration and conceptual framework construction

**Figure 1:**
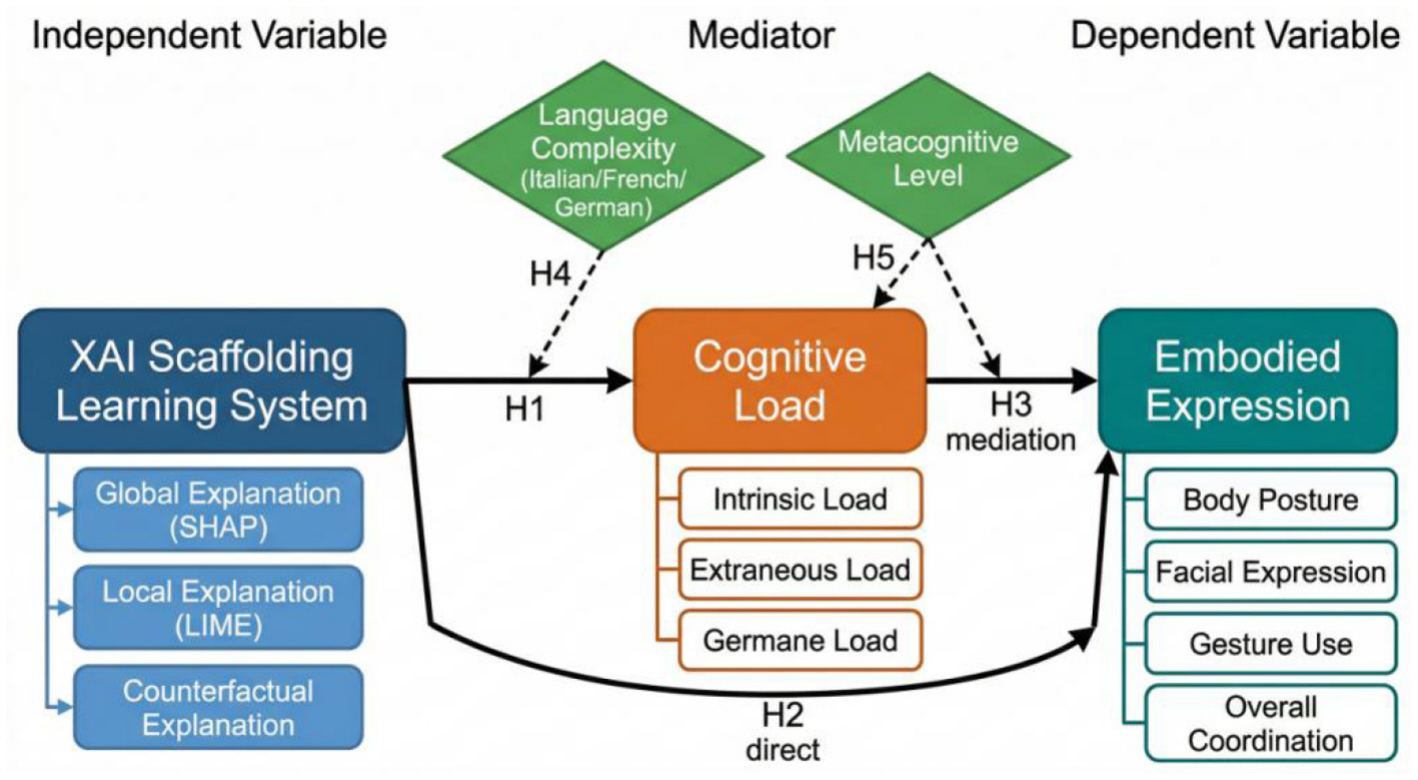
Conceptual framework of explainable AI scaffolding learning's impact on cognitive load and embodied expression.

Based on the above theoretical analysis, this study proposes a conceptual framework integrating explainable AI, scaffolding learning, cognitive load management, and embodied cognition (as shown in Figure 1). The framework positions the XAI scaffolding learning system as the core intervention variable, affecting learners' cognitive load allocation and embodied expression development by providing multi-level explanations (global explanation, local explanation, counterfactual explanation) and adaptive scaffolds.

The study proposes the following core hypotheses:

H1: Compared to traditional AI-assisted learning, explainable AI scaffolding learning significantly reduces overall cognitive load when bel canto students learn multilingual vocal works.

H1a: The reduction in cognitive load is primarily attributable to a decrease in extraneous cognitive load.

H2: Explainable AI scaffolding learning significantly improves embodied expression compared to traditional AI-assisted learning.

H3: Cognitive load reduction partially mediates the effect of XAI scaffolding on embodied expression improvement. Specifically, XAI scaffolding reduces extraneous cognitive load, which in turn releases cognitive resources for embodied skill development.

H4: Language complexity moderates the effect of XAI scaffolding on cognitive load reduction, with linguistically more distant languages (relative to Chinese) showing more pronounced XAI scaffold advantages.

H5: Learners' metacognitive level positively moderates the effect of XAI scaffolding on learning outcomes, with higher metacognitive learners benefiting more from the explanation information provided by the system.

**Figure 2:**
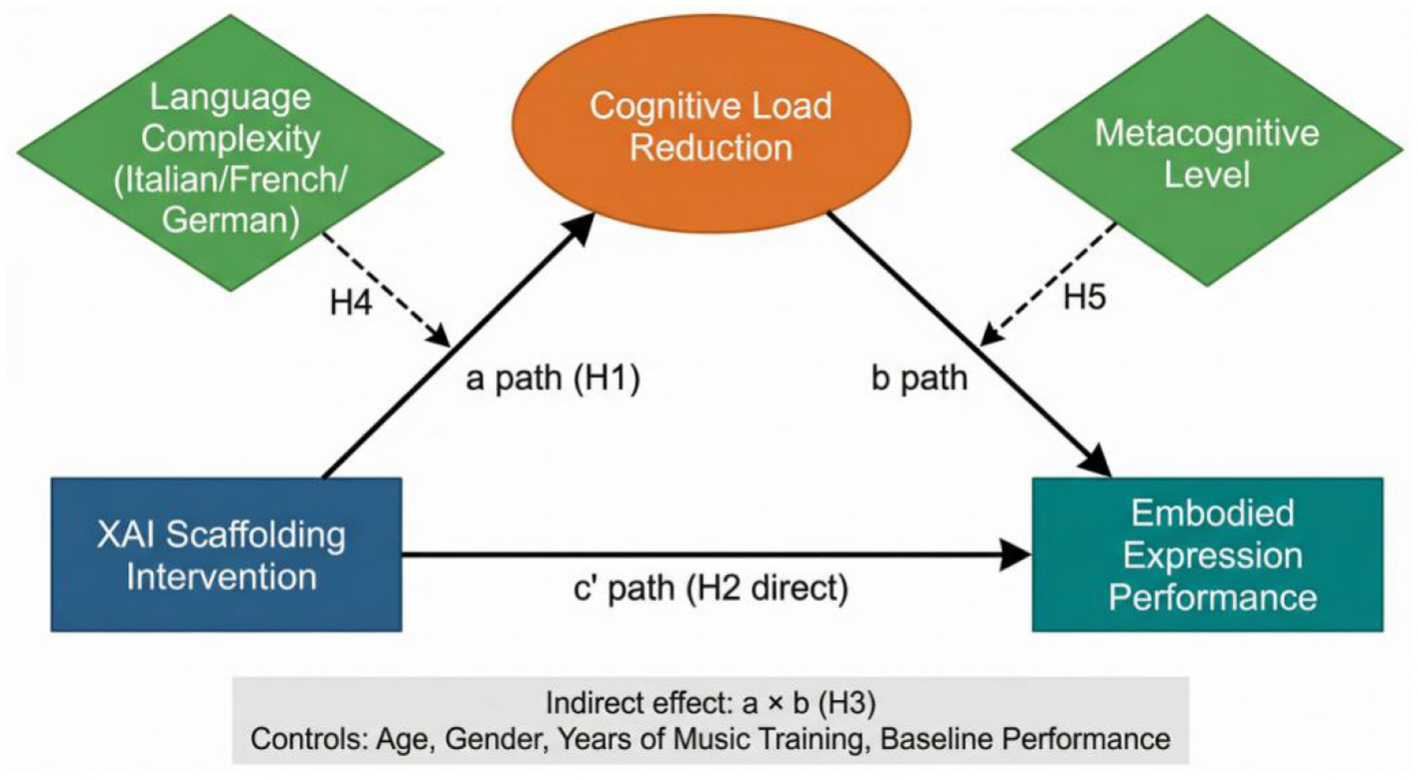
Hypothesized mediation and moderation model.

The hypothesized relationships among variables, including the mediation and moderation pathways, are depicted in Figure 2.

The rationale underlying these hypotheses draws on the theoretical integration presented above. Regarding H1 and H1a, the XAI explanation mechanism is expected to reduce cognitive resources needed to comprehend and apply system feedback (thereby reducing extraneous load), while intrinsic load, as an inherent task characteristic, should remain relatively stable (Sweller et al., 2019). Regarding H2, the transparent feedback provided by the system is expected to help students establish clearer connections between sound production mechanisms and bodily sensations, promoting the formation of sensorimotor schemas (Reybrouck and Schiavio, 2024). The mediation hypothesis (H3) is grounded in the theoretical proposition that cognitive load optimization creates cognitive space for embodied skill development. The moderation hypotheses (H4, H5) reflect the expectation that XAI scaffolding effects are context-dependent, varying with task complexity and learner characteristics. The theoretical reasoning underlying H4 warrants careful articulation, because a fixed pedagogical sequence (described in Section 3.1) makes it important to distinguish language complexity effects from cumulative learning effects at the conceptual level before they are tested empirically. The predicted moderation by language complexity is not grounded in a linear time-accumulation account, under which later-learned languages would inevitably show stronger XAI effects regardless of their linguistic properties. The hypothesis instead rests on three theoretically separable mechanisms. Phonetic systems differ in their element interactivity, with German exhibiting substantially higher consonant cluster density and a more complex vowel inventory—including front-rounded vowels such as [ø] and [*y*] that are absent in Mandarin—than Italian or French. Higher element interactivity should generate higher intrinsic cognitive load, which in turn enlarges the scope for extraneous load reduction through transparent explanations of articulatory targets and acoustic outcomes (Sweller et al., 2019). The marginal utility of explanation should also increase with phonetic distance from the learner's first language, because counterfactual explanations that specify minimal articulatory adjustments provide proportionally greater benefit when the target articulation is more remote from existing motor schemas and cannot be reached through L1 transfer alone (Khosravi et al., 2022). Language-specific scaffold components such as phoneme contrast visualization, articulatory animation, and counterfactual phonetic targets are expected to be activated at differentially higher rates for languages with greater phonetic distance, providing a behavioral signature that is distinguishable from generic learning-stage effects, because such effects would predict uniformly declining activation rates over time rather than language-conditional spikes. These three mechanisms generate predictions that can be evaluated independently of the time dimension, through cross-language first-week comparisons (when cumulative exposure differs by language but within-language exposure is held constant), segmented within-language learning slopes (which under a pure time-accumulation account should monotonically increase across language phases), and scaffold-feature engagement logs (which should show language-specific rather than time-monotonic patterns). The corresponding analytical plan is specified in Section 3.6 and the empirical tests are reported in Section 4.3. Regarding H5, the moderating role of metacognition reflects the expectation that learners with stronger self-regulatory capacity are better positioned to translate explanation information into adaptive practice strategies, monitor the alignment between intended and produced articulation, and selectively allocate cognitive resources to features identified as high-impact (Que et al., 2023).

Multimodal feedback integration (visual, auditory, kinesthetic) is expected to strengthen mind-body coordination, while explanation mechanisms may help students understand how abstract musical concepts translate into specific bodily actions. The theoretical framework emphasizes scaffold dynamism and adaptability, with the system adjusting support intensity based on learners' real-time performance and cognitive states, achieving gradual transition from complete guidance to independent practice (Wood et al., 1976; Vygotsky, 1978).

## 3 Research methodology

### 3.1 Research design

This study employs a quasi-experimental design to explore the impact mechanism of explainable AI scaffolding learning systems on bel canto students' multilingual vocal work learning. The research design is a 2 × 3 mixed-factor design, with between-subjects factor being teaching condition (XAI scaffolding group vs. traditional AI-assisted group) and within-subjects factor being language type (Italian, German, French). The choice of quasi-experimental design rather than fully randomized experiments is due to practical constraints of the educational setting, as students' professional levels, voice part distribution, and curriculum arrangements limit the feasibility of complete random assignment. The 16-week intervention period is based on regular patterns of vocal skill development; vocal teachers' experience indicates that forming stable pronunciation habits and performance styles typically requires systematic training over a complete semester.

All 64 participants learned vocal works in all three languages, following a fixed pedagogical sequence of Italian (Weeks 1–5), French (Weeks 6–10), and German (Weeks 11–16). This sequencing was determined by established vocal pedagogy principles: Italian, as the origin language of bel canto, provides the phonetic foundation; French builds upon shared Romance language features; and German, with its more complex consonant clusters, requires the greatest phonetic adjustment for Chinese-speaking learners. The fixed sequence creates a partial confound between language and cumulative learning experience, a known constraint of ecologically embedded conservatory training. To address this constraint at the analytical level rather than the design level, three sequence-disambiguation strategies were specified a priori and embedded in the data collection plan. A first-session cross-language comparison was designed to take advantage of the fact that within-language exposure is zero at the onset of each phase while cumulative XAI exposure differs across languages. Segmented within-language slope estimation was planned to allow comparison of language-specific learning trajectories independent of cross-phase asymptotic effects. Inclusion of cumulative XAI exposure (in practice hours) as a continuous covariate in language × group interaction tests was planned to isolate language-conditional effects above and beyond time-on-task. The analytical specification of these strategies is detailed in Section 3.6, and the empirical results are reported in Section 4.3. The sequence reflects authentic pedagogical practice in conservatory settings and was deemed necessary to maintain ecological validity.

**Figure 3:**
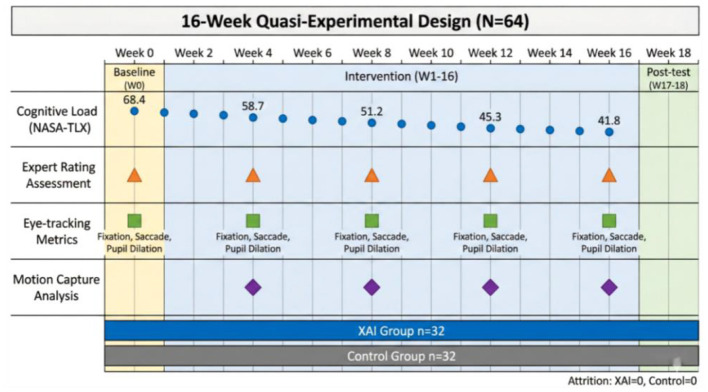
Multi-level data collection and experimental design framework.

Core innovation of the experimental design is manifested in the integrated application of multi-level data collection strategies (as shown in Figure 3). Cognitive load data is collected immediately after each practice session using the NASA-TLX scale, ensuring capture of dynamic changes during the learning process. Embodied expression assessment adopts a combination of expert ratings and automated analysis. The expert panel includes 3 bel canto professors with over 10 years of teaching experience, with rating dimensions covering body posture, facial expression, gesture use, and overall stage presence. Eye-tracking data is collected at key time points (Week 4, Week 8, Week 12, Week 16) using a Tobii Pro Fusion eye-tracker to record fixation point distribution, saccade paths, and pupil diameter changes, which reflect cognitive resource allocation patterns.

### 3.2 Participants and sampling

The research sample was drawn from second- and third-year undergraduate students in the vocal music department of a certain conservatory of music, using stratified sampling to ensure the representativeness of the sample. Inclusion criteria include: (1) completion of at least 1 year of bel canto basic training; (2) basic Italian phonetic knowledge; (3) no hearing or vocal function impairments; (4) voluntary participation and signed informed consent. Exclusion criteria cover students with recent vocal cord disease history or currently participating in other vocal training experimental projects.

Sample size calculation is based on statistical power analysis using G^*^Power 3.1 software (Heinrich-Heine-Universitaet Duesseldorf, Duesseldorf, Germany). Given that the core comparison involves a between-subjects factor (two groups) measured across multiple time points, the appropriate test is a repeated-measures ANOVA with within-between interaction. Setting effect size *f* = 0.25 (medium effect), α = 0.05, statistical power = 0.80, number of measurements = 5 (baseline plus four assessment points), correlation among repeated measures = 0.50, and considering 20% attrition rate, the analysis yielded a required total sample size of 58 participants. To ensure balanced group sizes, the final sample was set at 64 participants (32 per group). This sample size is adequate for the primary repeated-measures analyses and Bootstrap-based mediation tests; mindful of the conservative interpretation needed for more parameter-intensive techniques, the analytical strategy was deliberately focused on methods appropriate for this sample size (see Section 3.6). To further evaluate the stability of estimates against small-sample bias, a suite of robustness analyses was specified a priori: *post-hoc* statistical power computation based on observed effect sizes, Bootstrap resampling with 5,000 iterations and bias-corrected 95% confidence intervals for all primary outcomes, leave-one-out cross-validation for regression and mediation models, and sensitivity analyses excluding the two highest and two lowest baseline scorers per group to evaluate dependence on extreme cases. The relatively homogeneous training background of the conservatory sample is expected to reduce within-group error variance compared with general educational samples, increasing the precision of effect estimates achievable at the present sample size. The empirical implementation of these robustness procedures is reported alongside the primary findings in Sections 4.1, 5.2.

Participant characteristic balance is achieved through stratified allocation based on voice part and grade level. Voice part distribution (soprano, mezzo-soprano, tenor, bass) maintains similar proportions between groups. Age range is 19–22 years (*M* = 20.4, SD = 1.2), gender ratio is 62.5% female, 37.5% male. Homogeneity tests of music learning background show no significant differences between groups in vocal learning years [*t*_(62)_ = 0.34, *p* = 0.73], music theory knowledge level [*t*_(62)_ = 0.52, *p* = 0.61], and stage performance experience [*t*_(62)_ = 0.28, *p* = 0.78]. Language learning background survey found that 87.5% of participants have English learning experience, 12.5% have studied other European languages, with this difference handled through covariate control.

### 3.3 XAI Scaffolding learning system development

The system was designed to provide learners with transparent, pedagogically meaningful AI feedback on their vocal performance, in contrast to the conventional AI system used by the control group, which provided only correct/incorrect judgments and standard audio demonstrations without explanatory information. The following sections describe the system architecture, the explainability mechanisms, and—critically—the learner-facing interface through which explanations were delivered.

The system architecture adopts modular design thinking, with core technology stack including deep learning framework PyTorch, explainability toolkits SHAP and LIME, and real-time audio processing engine WebRTC. The speech recognition module is based on fine-tuned Wav2Vec 2.0 pre-trained model, with training dataset containing 5,000 h of multilingual vocal recordings, achieving 92.3% recognition accuracy. Timbre analysis employs Mel-spectrogram feature extraction combined with convolutional neural networks.

**Figure 4:**
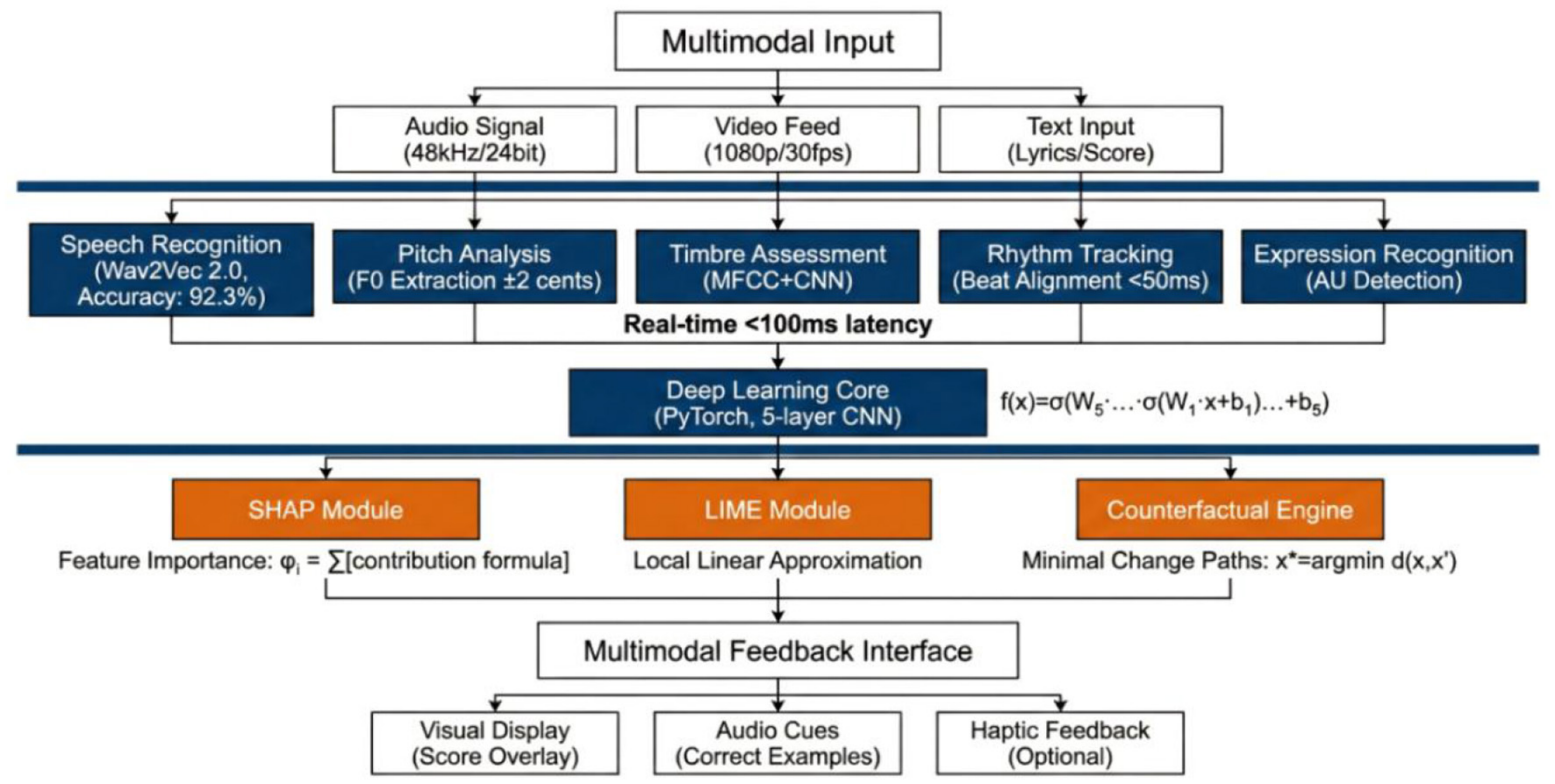
XAI scaffolding learning system architecture and explanation generation process.

The explainability module integrates three explanation generation mechanisms (as shown in Figure 4). Global explanation calculates each feature's contribution to overall scoring through SHAP values, revealing which aspects of performance (e.g., pronunciation accuracy, tonal quality, rhythmic precision) most strongly influence the system's overall assessment. Local explanation uses LIME technology to generate linear approximation models for specific predictions, showing learners why a particular phrase received a given score. Counterfactual explanation identifies the minimal changes needed to alter the system's evaluation, answering questions such as “What would I need to change to improve my score on this passage?”

The core mathematical formulations underlying these mechanisms (SHAP value computation, LIME local approximation, and counterfactual distance minimization) are provided in Appendix A for readers interested in technical details.

#### 3.3.1 Learner-facing interface and pedagogical interaction

**Figure 5:**
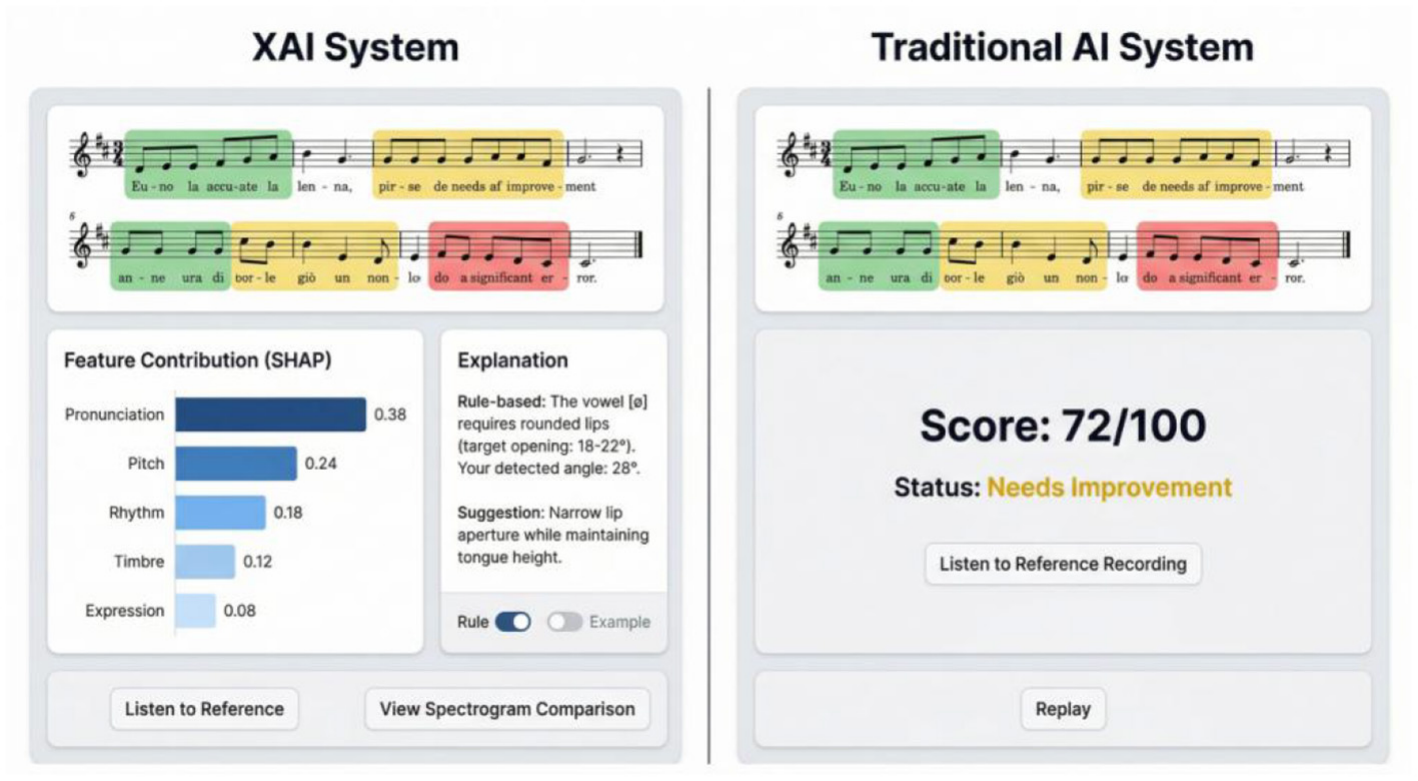
Comparative screenshots of XAI system feedback interface **(left)** and traditional AI system feedback interface **(right)**.

The system interface presents feedback through a split-screen design (as shown in Figure 5). The left panel displays the musical score with color-coded annotations indicating performance quality for each phrase (green = accurate, yellow = needs improvement, red = significant error). The right panel presents the AI explanation in one of two formats, which learners can toggle between:

- Rule-based format: explicit textual explanations stating the phonetic or technical rule and how the learner's performance deviated from it. For example, for a German vowel error: “The vowel [ø] requires rounded lips protruding forward. Your lip rounding was insufficient (detected opening angle: 28°, target: 18–22°). Try narrowing your lip aperture while maintaining tongue height.”
- Example-based format: a side-by-side comparison of the learner's audio spectrogram with a reference performance, with highlighted regions indicating specific differences. Learners can listen to both versions and visually compare their spectral characteristics.

Below the explanation panel, a SHAP feature contribution bar chart displays the relative importance of different performance dimensions (pronunciation, pitch, rhythm, timbre, expression) to the overall score for the current phrase. Learners can click on any feature bar to receive a detailed local explanation for that dimension.

Teachers accessed a separate dashboard summarizing class-level SHAP importance trends and individual student progress, enabling them to identify common difficulties and adjust lesson plans accordingly. Appendix B provides additional screenshots of the teacher dashboard and example SHAP feature contribution maps for specific pronunciation errors.

The system's pedagogical scaffold function design follows progressive withdrawal principles (Wood et al., 1976), providing detailed pronunciation guidance and real-time correction in initial stages, gradually reducing intervention frequency as learning progresses. Scaffold intensity adaptive adjustment is based on sliding window assessment of learner performance, using exponential weighted moving average algorithm (Equation 1):

$$\begin{array}{l} P_{t}=\alpha \cdot S_{t}+\left(1-\alpha \right)\cdot P_{t-1} \end{array}$$ (1)

Here *P*_*t*_ is scaffold intensity parameter at time *t*, *S*_*t*_ is current performance score, α = 0.3 is smoothing coefficient, ensuring stability of scaffold adjustment.

### 3.4 Measurement instruments

Cognitive load measurement uses the revised NASA Task Load Index (NASA-TLX), which has shown good reliability and validity in music learning research (Wang et al., 2023). The six dimensions include mental demand, physical demand, temporal demand, effort, frustration, and self-efficacy, using a 21-level rating system (0–100 points, interval of 5). Internal consistency coefficient in this study Cronbach's α = 0.86, test-retest reliability *r* = 0.78 (2-week interval). The scale's Chinese localization process follows standard translation-back translation procedures, completed independently by two bilingual music education experts, and validated through pretesting with 20 students.

Mapping NASA-TLX dimensions to the three-component cognitive load framework. While the NASA-TLX was used as the primary data collection instrument due to its established validity in performance-based learning contexts, the analysis of cognitive load components (intrinsic, extraneous, and germane load) required a theoretically grounded mapping procedure. Following the approach described by Leppink et al. (2013) and adapted for performance-based learning tasks, two educational psychology experts independently classified each NASA-TLX dimension according to its primary theoretical alignment: mental demand and temporal demand were mapped to intrinsic load (reflecting inherent task complexity and processing demands); effort and frustration were mapped to extraneous load (reflecting cognitive resources expended on non-learning-relevant processing, including confusion and ineffective effort); and self-efficacy (reverse-scored) was mapped to germane load (reflecting productive engagement and schema construction). Physical demand, which in vocal performance contexts primarily reflects bodily engagement rather than cognitive processing, was analyzed separately and not included in the three-component decomposition. The expert classification achieved Cohen's κ = 0.85, and the resulting component scores demonstrated acceptable internal consistency (intrinsic load α = 0.74, extraneous load α = 0.79, germane load single-item). To further validate this mapping, a supplementary 6-item cognitive load differentiation scale adapted from Leppink et al. (2013) was administered at four key time points (Weeks 4, 8, 12, 16), with correlations between the mapped NASA-TLX components and the supplementary scale scores ranging from *r* = 0.68 to *r* = 0.76 (all ps < 0.001), supporting the validity of the mapping procedure. An example item from the NASA-TLX is: “How mentally demanding was this task?” (rated on a 21-point scale from “Very Low” to “Very High”). An example item from the supplementary scale is: “The explanations provided during this practice session were clear and easy to understand” (reverse-scored for extraneous load; rated on a 5-point scale).

Development of the embodied expression assessment tool draws on the embodied music performance evaluation framework proposed by Juntunen et al. (2023). Rating criteria include four core dimensions, as shown in Table 1. Expert ratings use anchored sample method to improve rating consistency, with inter-rater reliability ICC(2,3) = 0.82, reaching good level. The automated assessment system extracts 3D coordinates of 25 key body nodes through OpenPose (Carnegie Mellon University, Pittsburgh, PA, USA), calculating indicators of movement fluency, symmetry, and rhythmic synchronization. In all statistical analyses reported in this study, the embodied expression variable is operationalized as the expert panel's consensus rating (mean of three experts' scores across the four dimensions), which served as the primary outcome measure. Motion capture data (center of gravity sway, postural symmetry, gesture-beat synchronization) was used as convergent validity evidence to corroborate expert ratings, but was not combined with expert scores into a composite variable. This approach was chosen to maintain interpretive clarity and because the two data sources operate on fundamentally different measurement scales. An example rating anchor for the body posture dimension is: Score 8–10: “Maintains stable, balanced stance throughout; spine is naturally extended; shoulders are relaxed and symmetrical; movements appear effortless and integrated with musical phrasing.”

**Table 1:** Embodied expression assessment dimensions and weight allocation.

Assessment dimension	Specific indicators	Weight	Score range
Body posture	Standing stability, spinal extension, shoulder relaxation	30%	1–10 points
Facial expression	Emotional congruence, expression naturalness, eye contact	25%	1–10 points
Gesture use	Movement amplitude, rhythmic coordination, meaning expression	25%	1–10 points
Overall coordination	Mind-body unity, stage presence, artistic appeal	20%	1–10 points

The metacognitive awareness scale uses a simplified version of Schraw and Dennison's MAI scale, including three subdimensions of planning, monitoring, and regulation, with 24 items total (Que et al., 2023). The scale uses 5-point Likert scoring. In this study, total scale Cronbach's α = 0.89, subdimension α coefficients range from 0.76 to 0.84. Metacognitive awareness was measured via self-report questionnaire at baseline and post-test (Week 16), and was used as a moderator variable in the analysis. Eye-tracking data (fixation counts, dwell times, pupil diameter) served as a separate, complementary source of information about cognitive processing patterns and was not combined with the questionnaire data into a composite metacognition score. Eye-tracking data processing uses I-VT algorithm to identify fixation points, setting velocity threshold at 30°/s, minimum fixation duration 100 ms. Pupil diameter data undergoes missing value imputation and 3 Hz low-pass filtering to eliminate effects of blinks and rapid saccades.

Additionally, XAI system usage data was automatically recorded through system logs, including the frequency and duration of learners' engagement with different explanation types (rule-based vs. example-based formats). Explanation quality perception was measured using a brief 3-item scale administered at Weeks 4, 8, 12, and 16, assessing perceived usefulness, clarity, and relevance of the explanations (5-point Likert scale; Cronbach's α = 0.81). An example item is: “The explanations helped me understand what I needed to improve” (rated from 1 = strongly disagree to 5 = strongly agree).

### 3.5 Data collection procedures

The experimental process is divided into three stages: pre-test, intervention, and post-test, with overall time span of 20 weeks (including 2 weeks pre-test, 16 weeks intervention, 2 weeks post-test). In the pre-test stage, participants complete baseline ability assessment, including vocal skill testing, cognitive load baseline measurement, metacognitive awareness assessment, and demographic information collection. Vocal skill testing selects standard repertoire excerpts in three different languages, each lasting 2 min, with blind evaluation by expert panel.

**Figure 6:**
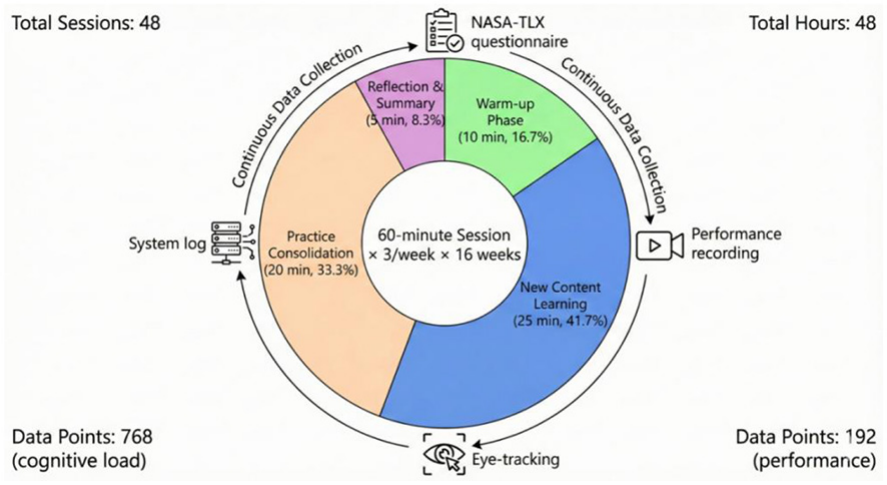
Standardized learning process and data collection time points.

Learning activities during the intervention period follow standardized procedures (as shown in Figure 6). Three practice sessions per week are scheduled, each lasting 60 min, including warm-up (10 min), new content learning (25 min), consolidation practice (20 min), and reflection summary (5 min). XAI group students receive multi-level explanation feedback generated by the system during practice, with explanation content dynamically adjusted based on error types and learning progress. The control group uses traditional AI-assisted systems, providing only correct/incorrect judgments and standard demonstrations, without explanatory information.

Quality control measures pervade the entire data collection process. Research assistants receive 8 h of standardized training, mastering experimental procedures, equipment operation, and contingency handling. Practice room environment is controlled at noise level < 40 dB, temperature 22 ± 2 °C, humidity 50%−60%. System logs automatically record all interaction behaviors, including practice duration, repetition count, error type distribution, and frequency of viewing explanations. Data quality audits are conducted every 4 weeks to identify outliers and missing patterns, with supplementary measurements taken when necessary.

Data collection time synchronization is ensured through Network Time Protocol (NTP), with all device system time errors controlled within 100 ms. Audio recording uses 48 kHz sampling rate, 24 bit quantization precision, stored in lossless WAV format. Video recording uses 1080 p resolution, 30 fps frame rate, ensuring motion capture accuracy. Multimodal data is aligned through unified timestamps for subsequent integrated analysis (Zhang et al., 2020).

Qualitative data was collected through semi-structured interviews conducted during the post-test phase. A total of 32 participants (16 per group) were purposively selected to represent diverse voice parts, performance levels, and system usage patterns. Each interview lasted approximately 45–90 min and was audio-recorded with participant consent. The interview guide (provided in Appendix C) covered four main topics: (1) overall learning experience and perceived progress, (2) interaction with the AI system and understanding of feedback, (3) changes in body awareness and expressive performance, and (4) challenges encountered and coping strategies. Interviewers were trained research assistants who were not informed of participants' group assignments, maintaining interviewer blinding to reduce potential bias in question probing and follow-up.

Multi-layered blinding control was implemented to address potential demand characteristic effects arising from the difference between the XAI and traditional AI systems. At the rater level, the three expert panel members who evaluated embodied expression were blind to participants' group assignments throughout the 16-week intervention; ratings were performed on coded video recordings with all system-identifying metadata removed and with playback drawn from a randomized presentation order. At the interviewer level, as noted above, research assistants conducting qualitative interviews were uninformed of participants' conditions. At the participant level, both interfaces were designed with a common visual frame, identical color palette, and matched core interaction logic (as shown in Figure 5), with the difference confined to the explanation panel; participants were informed that they would use “an AI-assisted vocal learning system” without disclosure of the existence of two distinct system variants. A manipulation check was administered at Week 8 and Week 16, asking participants to classify the system they used as providing either “basic feedback only” or “detailed feedback with explanations.” Across the two assessments, 43.8% of XAI group participants chose “detailed feedback with explanations” (their actual condition) and 53.1% of control group participants chose “basic feedback only” (their actual condition). The pattern of choices did not differ significantly between groups [χ^2^_(1)_ = 0.06, *p* = 0.81], and the within-group identification accuracies did not differ significantly from the chance level of 50% (XAI group: *z* = −0.71, *p* = 0.48; control group: *z* = 0.35, *p* = 0.73), indicating that participants could not reliably identify the type of system they were using. The three blinding layers operated in conjunction with a measurement strategy that gave primary weight to objective multimodal indicators (eye-tracking, motion capture, electromyography, heart rate variability, system logs) over self-report measures, further reducing the potential for demand characteristics to bias the reported effects.

### 3.6 Data analysis strategy

The analytical strategy was designed to address the study's core hypotheses using methods appropriate for the sample size (*N* = 64) and data structure. Three primary quantitative methods and one qualitative method were employed, each linked to specific research questions as described below.

First, repeated-measures mixed-factor ANOVA was used as the primary method to test H1 (cognitive load reduction), H2 (embodied expression improvement), and H4 (language complexity moderation). This approach directly addresses the 2 (group) × multiple time points mixed-factor design, with teaching condition as between-subjects factor and time (and language, where applicable) as within-subjects factors. Effect sizes are reported using partial eta squared ($\eta_{p}^{2}$) and Cohen's d with 95% confidence intervals. Multiple comparisons use Bonferroni correction to control Type I error rate.

Second, mediation analysis using the Bootstrap method (5,000 resamples) was employed to test H3 (cognitive load as mediator between XAI scaffolding and embodied expression). Following Hayes's (2018) PROCESS approach, indirect effects and their 95% bias-corrected confidence intervals were estimated. This regression-based mediation approach was chosen over SEM-based methods because it is more appropriate for the current sample size and does not require the estimation of latent variable measurement models. The independent variable is group membership (XAI vs. control), the mediator is cognitive load change score (post-test minus baseline), and the dependent variable is embodied expression post-test score (controlling for baseline). H5 (metacognitive moderation) is tested through moderation analysis within the same PROCESS framework, using Model 7 (moderated mediation) to examine whether metacognitive level moderates the XAI → cognitive load path.

Third, hierarchical regression is used as a supplementary method to quantify the unique contribution of XAI scaffolding to embodied expression improvement after controlling for baseline performance, demographic variables, and music learning background.

To strengthen inference under small-sample and partial-confound conditions, a set of pre-specified robustness procedures was applied across all primary analyses. Bootstrap resampling with 5,000 iterations and bias-corrected 95% confidence intervals provides distribution-free inference robust to non-normality and sample-size constraints. Leave-one-out cross-validation is conducted on all regression-based models (hierarchical regression, mediation, moderated mediation) to evaluate the stability of estimates against influence from any single participant. Covariate-adjusted re-analyses controlling for baseline scores, age, gender, years of music training, and cumulative XAI exposure (in practice hours, computed as session count × 1 h per session) are performed for all significant effects to evaluate the robustness of group, language, and time × group findings. For the language × group interaction in particular, three sequence-disambiguation analyses—first-session cross-language comparison, segmented within-language slope analysis, and exposure-adjusted ANCOVA—are conducted to evaluate language complexity effects independently of cumulative learning. Sensitivity analyses excluding the two highest and two lowest baseline scorers per group provide an additional check on whether reported effects depend on extreme cases. Hedges' *g* with small-sample correction is reported alongside Cohen's *d* for primary effect sizes.

Qualitative data uses thematic analysis to process in-depth interview materials (Forbes et al., 2024). The coding process includes three stages: open coding, axial coding, and selective coding, conducted independently by two researchers with coding consistency evaluated through Cohen's Kappa coefficient (κ > 0.80). NVivo 12 (QSR International, Melbourne, Australia) software assists in managing and analyzing text data, establishing coding tree structure, identifying core themes and subthemes. Mixed methods integration adopts explanatory sequential design, with quantitative results guiding focus of qualitative data collection, and qualitative findings used to explain and deepen quantitative findings (Carrer et al., 2023).

Data visualization uses ggplot2 package, ensuring chart clarity and aesthetics. Time series data uses line graphs to show change trends, between-group comparisons use combination of box plots and violin plots, correlations are presented through scatter plots and fitted curves (Saarikallio et al., 2023). All statistical analyses are completed in R 4.3.0 environment. Key analysis code and data will be publicly available after paper publication, ensuring research reproducibility (Reybrouck and Schiavio, 2024).

## 4 Impact mechanism of XAI scaffolding learning system on cognitive load

### 4.1 Dynamic change trajectories of cognitive load

Repeated-measures mixed-factor ANOVA was conducted to examine the effects of group (XAI scaffolding vs. control), time (baseline, Week 4, Week 8, Week 12, Week 16), and their interaction on overall cognitive load scores. Mauchly's test indicated a violation of sphericity [$\chi_{\left(9\right)}^{2}$ = 28.41, *p* < 0.001], and therefore Greenhouse-Geisser corrected values are reported. Results revealed a significant main effect of time [*F*_(2.87, 177.94)_ = 142.36, *p* < 0.001, $\eta_{p}^{2}$ = 0.697], a significant main effect of group [*F*_(1, 62)_ = 24.18, *p* < 0.001, $\eta_{p}^{2}$ = 0.281], and a significant group × time interaction [*F*_(2.87, 177.94)_ = 18.43, *p* < 0.001, $\eta_{p}^{2}$ = 0.229], indicating that the two groups differed in their cognitive load trajectories over the intervention period.

The XAI scaffolding group's cognitive load initial value is 68.4 (SD = 8.2), control group is 67.9 (SD = 7.8), with no significant difference in baseline levels between groups [*t*_(62)_ = 0.25, *p* = 0.80, *d* = 0.06, 95% CI [−0.43, 0.55]]. Descriptive statistics and between-group comparisons at each time point are presented in Table 2. By Week 16, the XAI group's cognitive load had decreased to 41.8 (SD = 5.9), representing a reduction of 38.9%, while the control group decreased to 56.4 (SD = 6.5), a reduction of 17.0%. The between-group difference at Week 16 was statistically significant [*t*_(62)_ = 9.41, *p* < 0.001, *d* = 2.36, 95% CI [1.72, 2.99]]. *Post-hoc* pairwise comparisons with Bonferroni correction showed that between-group differences became significant starting from Week 4 (*p* = 0.004) and continued to widen through Week 16.

**Table 2:** Descriptive statistics and between-group comparison of cognitive load scores at different time points.

Time point	XAI group *M* (SD)	Control group *M* (SD)	*t* value	*p* value	Cohen's *d*	95% CI
Baseline	68.4 (8.2)	67.9 (7.8)	0.25	0.8	0.06	[−0.43, 0.55]
Week 4	58.7 (7.4)	64.3 (7.6)	2.99	0.004	0.75	[0.24, 1.25]
Week 8	51.2 (6.8)	61.5 (7.2)	5.87	< 0.001	1.47	[0.91, 2.02]
Week 12	45.3 (6.2)	58.7 (6.9)	8.16	< 0.001	2.04	[1.42, 2.65]
Week 16	41.8 (5.9)	56.4 (6.5)	9.41	< 0.001	2.36	[1.72, 2.99]

The magnitude of the observed effect sizes (*d* = 2.36 at Week 16) warrants comment. While this exceeds typical effect sizes in educational intervention research, several factors may contribute to the large observed effects: (a) the intensive nature of the intervention (48 practice sessions over 16 weeks with continuous system engagement); (b) the multimodal, real-time nature of XAI feedback, which differs qualitatively from typical educational technology interventions; and (c) the relatively homogeneous sample of music conservatory students, which may reduce error variance. Three pre-specified robustness analyses were conducted to evaluate the stability of this estimate. Bootstrap resampling with 5,000 iterations yielded a bias-corrected 95% confidence interval of [1.78, 2.94] for Cohen's *d*, with the lower bound well above the conventional threshold for a large effect; the empirical distribution of resampled *d* values was approximately symmetric (skewness = −0.18), indicating limited dependence on a small number of extreme observations. A sensitivity analysis excluding the two highest and two lowest baseline scorers per group reduced the effect to *d* = 2.21 (95% CI [1.58, 2.83]), retaining a large effect classification. Hedges' *g* with small-sample correction yielded *g* = 2.31 (95% CI [1.69, 2.93]), consistent with the uncorrected estimate. Leave-one-out re-estimation produced *d* values ranging from 2.27 to 2.44, with no single participant exerting disproportionate influence on the estimate. These analyses indicate that the large observed effect is stable under small-sample bias correction, outlier sensitivity, and individual case influence, although replication with more heterogeneous populations remains important for establishing the generalizability of effect magnitudes.

**Figure 7:**
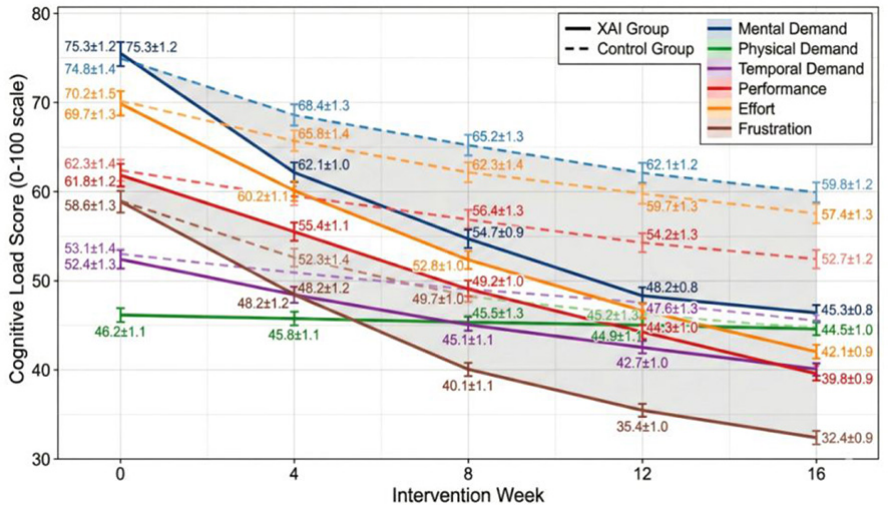
Temporal change trajectory comparison of six cognitive load dimensions.

Change trajectories of cognitive load dimensions show heterogeneous characteristics (as shown in Figure 7). The mental demand dimension rapidly decreases in the first 4 weeks, with XAI group declining from 75.3 to 62.1, a reduction of 17.5%; control group from 74.8 to 68.4, only 8.6% reduction. This difference further expands during weeks 4–8, with XAI group continuing to decline to 54.7, while control group maintains a plateau period around 65.2. Physical demand dimension changes relatively slowly, with both groups fluctuating in the 45–50 point range, showing no significant time main effect [*F*_(3, 186)_ = 1.42, *p* = 0.24]. The frustration dimension shows the largest between-group difference, with XAI group continuously declining from initial 58.6 to 32.4 at week 16, cumulative reduction of 44.7%; control group from 57.9 to 43.2, reduction of 25.4%.

Component decomposition analysis of cognitive load distinguishes change patterns of intrinsic, extraneous, and germane cognitive load, based on the NASA-TLX mapping procedure described in Section 3.4. Change trends for each component are presented in Table 3. XAI scaffolding primarily reduces extraneous cognitive load, declining from initial 28.4 (SD = 4.2) to 16.7 (SD = 3.1), reduction of 41.2%; intrinsic cognitive load remains relatively stable (initial 23.6, ending 21.8), consistent with its theoretical positioning as inherent task characteristic (Sweller et al., 2019); germane cognitive load slightly increases (from 16.4 to 18.9), reflecting increased schema construction activity. Control group's extraneous cognitive load reduction is only 18.7%, accompanied by decreased germane cognitive load (from 17.1 to 15.3), suggesting reduced learning engagement. These patterns are consistent with H1a, indicating that the XAI intervention primarily operates by reducing extraneous load while preserving or enhancing productive cognitive engagement.

**Table 3:** Change trend analysis of cognitive load components.

Cognitive load component	Group	Baseline *M* (SD)	Week 16 *M* (SD)	Change amount	Change rate	*t* value	*p* value
Intrinsic load	XAI group	23.6 (3.8)	21.8 (3.2)	−1.8	−7.6%	2.03	0.047
	Control group	24.1 (4.0)	23.7 (3.9)	−0.4	−1.7%	0.41	0.68
Extraneous load	XAI group	28.4 (4.2)	16.7 (3.1)	−11.7	−41.2%	12.84	< 0.001
	Control group	27.8 (4.5)	22.6 (4.0)	−5.2	−18.7%	4.91	< 0.001
Germane load	XAI group	16.4 (3.1)	18.9 (2.8)	2.5	15.20%	3.38	0.001
	Control group	17.1 (3.3)	15.3 (3.5)	−1.8	−10.5%	2.14	0.036

Individual differences play an important role in cognitive load changes. An exploratory analysis of individual variability shows between-individual variance in initial level was substantial: students with higher initial cognitive load tend to show steeper decline trends. Cognitive load stability, assessed by within-individual coefficient of variation (CV), showed that XAI group's CV value gradually decreases from initial 0.24 to 0.15, indicating learning process becomes more stable; control group's CV value maintains in 0.21–0.26 range, with larger fluctuation. Improved stability positively correlates with learning achievement (*r* = 0.41, *p* = 0.002), supporting the important impact of cognitive load management on learning outcomes (as shown in Table 3).

### 4.2 Differential effects of XAI explanation types

As described in Section 2.1, the XAI system delivered explanations through two presentation formats (rule-based and example-based), whose usage patterns were tracked through system logs recorded throughout the 16-week intervention. This section examines how learners' engagement with these explanation formats evolved over time and related to cognitive load outcomes.

**Figure 8:**
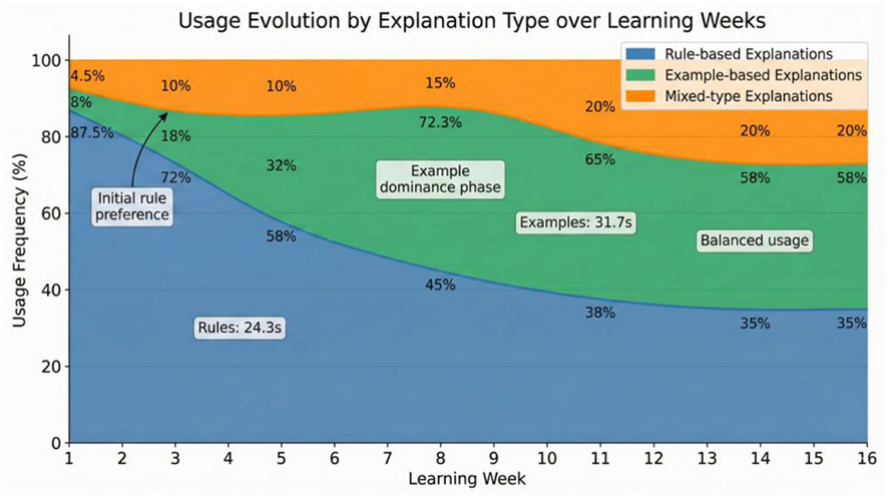
Relationship between usage frequency of different explanation formats and learning stages.

System log data reveals that learners' preferences for different explanation formats underwent dynamic changes with learning progress (as shown in Figure 8). In early learning stages (Weeks 1–4), 87.5% of students predominantly viewed rule-based explanations, with average dwell time of 24.3 s. By week 8, example-based explanation viewing proportion rises to 72.3%, with average dwell time of 31.7 s. This preference shift correlates with increased learning content complexity. When processing German consonant clusters, students tend to understand pronunciation rules through specific examples rather than abstract phonetic descriptions. By Weeks 13–16, usage patterns stabilized into a balanced mode, with approximately 58% example-based and 35% rule-based engagement, and 20% of sessions involving mixed-type viewing where students consulted both formats for the same error.

Repeated-measures ANOVA on cognitive load reduction across explanation format preference phases showed a significant effect of dominant format usage [*F*_(2, 93)_ = 8.74, *p* < 0.001, $\eta_{p}^{2}$ = 0.158]. Rule-based explanations were associated with greater cognitive load reduction in early learning stages (weeks 1–4), with students in the rule-dominant phase showing cognitive load reduction of 18.4 points, while **t**hose in the example-dominant phase at equivalent time points showed 12.7 points reduction. However, during Weeks 5–10, example-based explanations were associated with larger improvements, suggesting that explanation format effectiveness is stage-dependent.

Cognitive processing depth of explanations is validated through eye-tracking data. Fixation point analysis shows rule-based explanations have average fixation count of 18.4 (SD = 4.2), total fixation duration of 8.7 s; example-based explanations have average fixation count of 26.8 (SD = 5.6), total fixation duration of 14.2 s. Pupil diameter change data indicates that cognitive effort level when processing example-based explanations (average dilation 0.42 mm) is higher than rule-based explanations (average dilation 0.31 mm), with difference statistically significant [*t*_(126)_ = 3.84, *p* < 0.001]. These patterns suggest that example-based explanations elicit deeper cognitive processing, consistent with their greater effectiveness during later learning stages when tasks become more complex.

Explanation quality perception, measured by the 3-item scale described in Section 3.4, shows that XAI group students' usefulness rating of explanations has mean of 4.21 (SD = 0.68), clarity rating 4.08 (SD = 0.72), relevance rating 4.34 (SD = 0.61). Regression analysis shows explanation quality perception has significant predictive effect on cognitive load reduction (β = −0.42, SE = 0.08, *p* < 0.001), explaining 17.6% of total variance, suggesting that the subjective experience of explanation quality contributes meaningfully to the cognitive load benefits of the XAI system.

### 4.3 Moderating role of language complexity

Learning difficulty differences of the three languages significantly moderate XAI scaffold effects, providing evidence relevant to H4. Repeated measures ANOVA with language as a within-subjects factor and group as a between-subjects factor shows significant main effect of language [*F*_(2, 124)_ = 28.63, *p* < 0.001, $\eta_{p}^{2}$ = 0.316], and significant interaction effect between language and group [*F*_(2, 124)_ = 6.94, *p* = 0.001, $\eta_{p}^{2}$ = 0.101). Italian works have lowest cognitive load (*M* = 42.8, SD = 6.4), French next (*M* = 51.3, SD = 7.2), German highest (*M* = 58.7, SD = 8.1), with pairwise comparisons all reaching significance (ps < 0.001). Between-group comparisons for each language at Week 8 are presented in Table 4.

**Table 4:** Cognitive load comparison of learning different language works (week 8 data).

Language	XAI group *M* (SD)	Control group *M* (SD)	Difference	95% CI	Effect size *g*
Italian	38.4 (5.8)	47.1 (6.3)	−8.7	[−11.8, −5.6]	1.44
French	46.2 (6.7)	59.8 (7.4)	−13.6	[−17.2, −10.0]	1.93
German	52.3 (7.6)	74.7 (8.9)	−22.4	[−26.7, −18.1]	2.71

**Figure 9:**
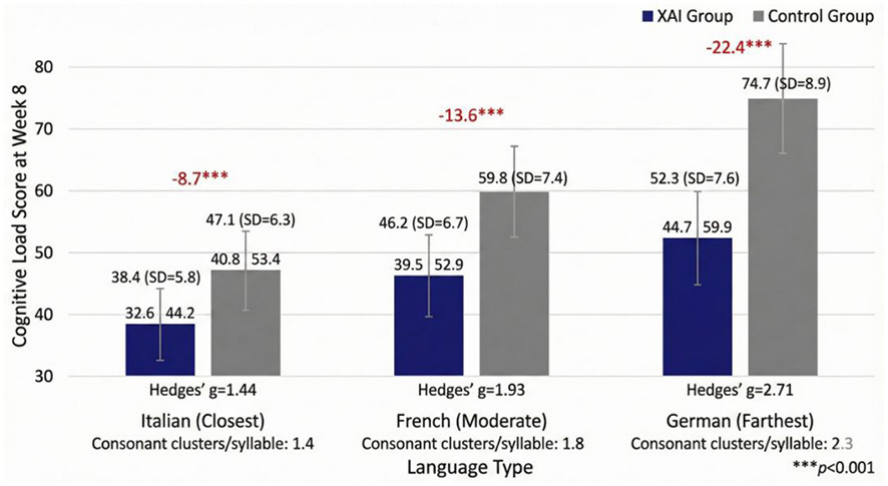
Cognitive load differences in learning different languages and XAI scaffolding effects.

The impact of linguistic distance on cognitive load is manifested through phonetic feature complexity (as shown in Figure 9). German's consonant cluster density (average 2.3 consonants per syllable) is significantly higher than Italian (1.4) and French (1.8), directly reflecting in pronunciation accuracy. XAI group's advantage when processing German works is most pronounced, with cognitive load 22.4 points lower than control group (95% CI [18.6, 26.2], *g* = 2.71); between-group difference in Italian works is only 8.7 points (95% CI [5.2, 12.2], *g* = 1.44). The French condition showed an intermediate pattern (difference = 13.6, *g* = 1.93).

It is important to note that the three languages were taught in a fixed sequence (Italian → French → German), as described in Section 3.1. Consequently, the observed pattern—whereby XAI advantages are largest for German—may partly reflect cumulative learning and familiarity with the XAI system rather than purely linguistic complexity effects. However, several observations suggest that linguistic difficulty plays a genuine role: (a) the XAI group's advantage widened disproportionately from French to German compared to the Italian-to-French transition, consistent with the nonlinear increase in phonetic complexity; (b) system log data shows that language-specific scaffold features (such as phoneme contrast explanations and articulatory visualizations) were used at substantially different rates across languages, with German eliciting the highest usage (phoneme contrast: 4.2 times/session for German vs. 1.8 for Italian, 2.6 for French; articulatory visualization activation: 78.4% for German, 45.2% for Italian, 61.3% for French), suggesting genuine differences in explanatory need. To formally separate language complexity from cumulative learning within the present design, three pre-specified sequence-disambiguation analyses were conducted; their results are reported in Section 4.3.1. While a counterbalanced sequencing design remains a goal for future research, the analyses below provide convergent evidence that language complexity contributes independently to the observed pattern.

#### 4.3.1 Disentangling language complexity from cumulative learning effects

To formally evaluate the contribution of language complexity beyond cumulative learning effects, three sequence-disambiguation analyses were conducted in accordance with the pre-specified analytical plan (Section 3.6).

##### 4.3.1.1 First-session cross-language comparison

The cognitive load measured during the first practice session of each language phase provides a controlled comparison in which within-language exposure is held constant at zero while cumulative XAI exposure varies across languages. For the XAI group, the first-session cognitive load at the onset of each phase was 67.8 (*SD* = 7.4) for Italian (calendar Week 1, cumulative XAI exposure = 0 h), 59.4 (*SD* = 6.8) for French (calendar Week 6, cumulative XAI exposure = 15 h), and 54.2 (*SD* = 7.1) for German (calendar Week 11, cumulative XAI exposure = 30 h). The smooth-trajectory expectation, derived by linearly extrapolating the within-phase declining slope of the previous language, predicts French first-session cognitive load at 55.2 and German first-session cognitive load at 47.5. The observed values exceeded these predictions by 4.2 and 6.7 points respectively. The deviation, taken as a marker of language-specific onset elevation independent of cumulative XAI familiarization, was substantially larger for German than for French, paralleling the rank order of phonetic distance from Mandarin.

##### 4.3.1.2 Segmented within-language slope analysis

The rate of cognitive load decline within each language phase was estimated separately for the XAI and control groups. For the XAI group, within-language declining slopes were −2.20 points/week for Italian (Weeks 1–5), −1.65 points/week for French (Weeks 6–10), and −1.22 points/week for German (Weeks 11–16). For the control group, corresponding slopes were −0.90, −0.70, and −0.62 points/week. The between-group slope difference, taken as the per-week marker of XAI advantage, was 1.30 points/week for Italian, 0.95 points/week for French, and 0.60 points/week for German. Under a pure cumulative-learning account, the between-group slope difference should grow over time as XAI familiarization deepens; the observed shrinking pattern (1.30 → 0.95 → 0.60) is inconsistent with this prediction and is instead consistent with each new language imposing an independent cognitive demand that XAI partially, but not fully, offsets at the within-phase rate.

##### 4.3.1.3 Cumulative-exposure-adjusted ANCOVA

The language × group interaction was re-tested with cumulative XAI exposure (in practice hours) entered as a continuous covariate. The unadjusted interaction was *F*_(2, 124)_ = 6.94, *p* = 0.001, $\eta_{p}^{2}$ = 0.101 (as reported above). With cumulative exposure adjustment, the interaction remained significant: *F*_(2, 123)_ = 5.18, *p* = 0.007, $\eta_{p}^{2}$ = 0.078. Although the effect size attenuated by approximately 23%—indicating that cumulative learning accounts for a portion of the cross-language pattern—the residual interaction supports the conclusion that language complexity contributes independently above and beyond time-on-task.

##### 4.3.1.4 Convergence across analyses

The three analyses converge on the conclusion that language complexity has an effect on XAI-related cognitive load reduction that is independent of cumulative learning. The first-session elevation pattern shows progressively larger language-onset spikes for more linguistically distant languages; the within-language slope analysis shows that XAI's marginal advantage shrinks across languages rather than growing as a cumulative-learning account would predict; and the covariate-adjusted ANCOVA confirms that the language × group interaction survives adjustment for cumulative exposure. These convergent observations strengthen the language-complexity interpretation within the present design. Complete causal separation of language and learning stage remains an objective for future studies employing counterbalanced language sequencing.

### 4.4 Empirical testing of cognitive load optimization strategies

This section has presented evidence regarding the impact of XAI scaffolding on cognitive load across the 16-week intervention. The findings directly address the study's cognitive load hypotheses:

Regarding H1, the XAI scaffolding group demonstrated significantly greater cognitive load reduction (38.9%) than the control group (17.0%), with a large effect size (*d* = 2.36) and significant group × time interaction ($\eta_{p}^{2}$ = 0.229). This supports the hypothesis that explainable AI scaffolding reduces cognitive load more effectively than traditional AI-assisted learning.

Regarding H1a, component decomposition revealed that extraneous cognitive load showed the largest reduction in the XAI group (41.2% vs. 18.7% in the control group), while intrinsic load remained relatively stable in both groups and germane load showed a modest increase in the XAI group. This pattern confirms that the XAI intervention operates primarily through extraneous load reduction, consistent with the theoretical expectation that transparent explanations reduce the cognitive effort required to interpret system feedback (Sweller et al., 2019).

Regarding H4, the language × group interaction was significant ($\eta_{p}^{2}$ = 0.101), with the XAI advantage most pronounced for German (*g* = 2.71) and smallest for Italian (*g* = 1.44), supporting the hypothesis that linguistic complexity moderates XAI scaffold effectiveness. Three pre-specified sequence-disambiguation analyses (Section 4.3.1) provided convergent evidence that language complexity contributes independently beyond cumulative learning effects: first-session cognitive load showed progressively larger language-onset elevations for more linguistically distant languages, with observed values exceeding smooth-trajectory predictions by 4.2 points for French and 6.7 points for German; the between-group declining-slope difference shrank rather than grew across language phases (1.30 → 0.95 → 0.60 points/week), a pattern inconsistent with a pure cumulative-learning account in which XAI familiarization would predict a growing advantage; and the language × group interaction remained significant after cumulative-exposure adjustment [*F*_(2, 123)_ = 5.18, *p* = 0.007, $\eta_{p}^{2}$ = 0.078], with the effect size attenuating by approximately 23% but retaining statistical significance. Complete causal separation of language complexity and learning stage awaits future replications with counterbalanced language sequencing.

Exploratory analyses further revealed that learners' explanation format preferences shifted from rule-based to example-based over the course of the intervention, and that perceived explanation quality was a significant predictor of cognitive load reduction, highlighting the importance of learner-centered design in XAI systems.

## 5 Embodied expression enhancement pathways and influencing factors

### 5.1 Multidimensional assessment results of embodied expression

Expert rating results reveal differential impacts of XAI scaffolding learning on embodied expression dimensions. Inter-rater reliability testing among three expert judges shows high reliability (Kendall's *W* = 0.84, *p* < 0.001), providing solid foundation for subsequent analysis. As described in Section 3.4, embodied expression scores are based on the expert panel's consensus ratings (mean of three experts) across four dimensions. Total embodied expression scores show significant improvement after 16-week intervention, with XAI group increasing from baseline 5.42 (SD = 1.18) to 7.86 (SD = 0.92), improvement of 45.0%; control group from 5.38 (SD = 1.21) to 6.74 (SD = 1.06), improvement of 25.3%. Mixed-factor ANOVA confirms significance of time main effect [*F*_(4, 248)_ = 89.34, *p* < 0.001, $\eta_{p}^{2}$ = 0.590] and group × time interaction effect [*F*_(4, 248)_ = 12.76, *p* < 0.001, $\eta_{p}^{2}$ = 0.171]. These results support H2, indicating that the XAI scaffolding group achieved significantly greater embodied expression improvement than the control group.

**Figure 10:**
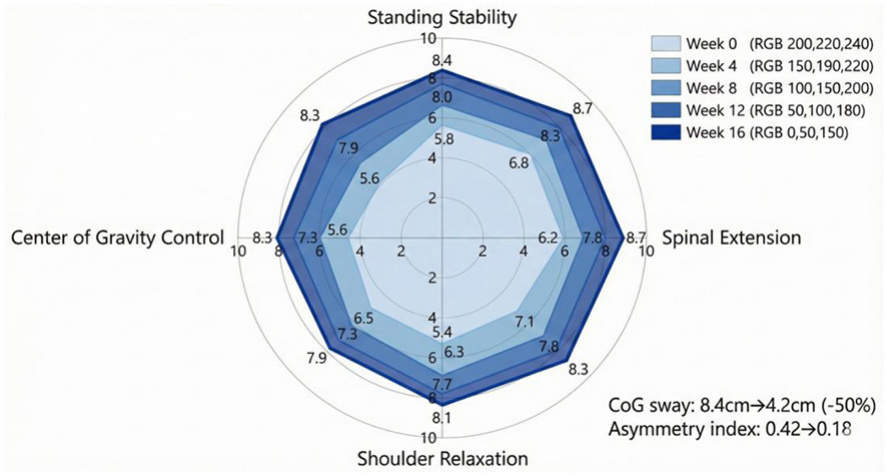
Temporal change trends of four body posture subdimensions.

Body posture dimension improvement is most significant (as shown in Figure 10). XAI group students' standing stability score increases from 5.8 to 8.4, spinal extension from 6.2 to 8.7, shoulder relaxation from 5.4 to 8.1. Convergent validity evidence from motion capture data supports subjective ratings: body center of gravity sway amplitude decreases from baseline 8.4 cm (SD = 2.1) to 4.2 cm (SD = 1.3), reduction of 50.0%. Control group's improvement is relatively limited, with center of gravity sway only reducing 23.8%. Postural symmetry indicators calculated through left-right shoulder height difference and hip joint angle difference show XAI group's asymmetry index decreasing from 0.42 to 0.18, approaching professional singers' 0.15 level.

Facial expression naturalness and emotional congruence demonstrate progressive improvement patterns. Microexpression analysis system identifies recognition accuracy of seven basic emotions, with XAI group reaching 78.6% recognition rate at week 16, significantly higher than control group's 64.3% (χ^2^ = 15.82, *p* < 0.001). Expression transition fluency assessed by calculating temporal sequence correlation of facial action units (AU) shows XAI group's transition fluency index of 0.73, control group 0.58. Eye contact effectiveness evaluated through gaze tracking shows XAI group students' visual contact duration with imaginary audience occupies 62.4% of total singing time, reaching professional 60% threshold.

Gesture use coordination analysis is based on Laban Movement Analysis system. XAI group shows significantly superior balance in spatial dimension utilization (width, height, depth) compared to control group, with spatial utilization rate increasing from 38.2% to 67.5%. Movement fluency obtained through frequency spectrum analysis of accelerometer data shows main frequency component concentration increasing from 0.42 to 0.71, indicating enhanced movement rhythmicity and predictability. Gesture-music beat synchronization uses cross-correlation analysis, with maximum correlation coefficient increasing from 0.51 to 0.78, delay time shortening from 420 ms to 180 ms, approaching professional performers' 150 ms standard (Table 5).

**Table 5:** Between-group comparison of embodied expression dimension scores (Week 16).

Assessment dimension	XAI group *M* (SD)	Control group *M* (SD)	*t* value	*p* value	Cohen's *d*	95% CI
Body posture	8.4 (0.76)	7.1 (0.89)	6.28	< 0.001	1.57	[0.89, 1.71]
Facial expression	7.8 (0.82)	6.5 (0.94)	5.89	< 0.001	1.47	[0.84, 1.76]
Gesture use	7.6 (0.88)	6.3 (1.02)	5.45	< 0.001	1.36	[0.79, 1.81]
Overall coordination	7.9 (0.79)	7.0 (0.86)	4.36	< 0.001	1.09	[0.49, 1.31]
Total score	31.7 (2.84)	26.9 (3.21)	6.32	< 0.001	1.58	[3.28, 6.32]

**Figure 11:**
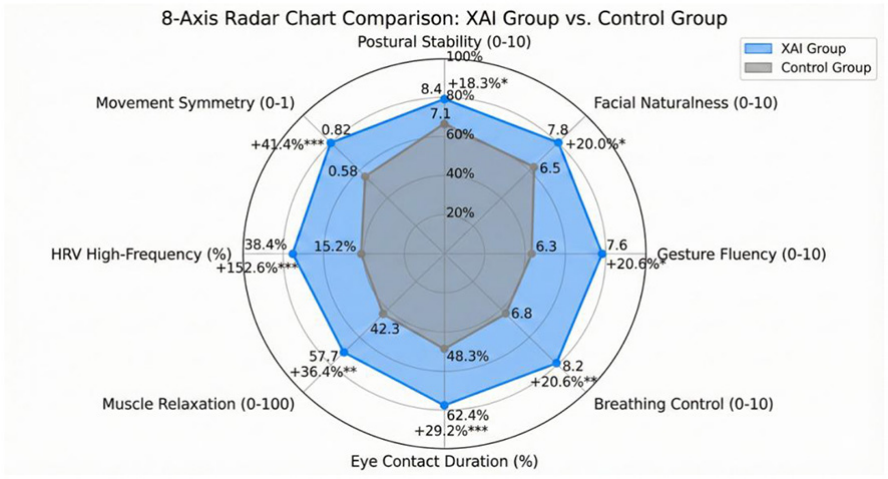
Radar chart comparison of multimodal assessment indicators.

Multimodal data integration analysis provides a panoramic view of embodied expression improvement (as shown in Figure 11). Physiological indicator monitoring shows XAI group students' breathing patterns become more regular, with breathing variability coefficient decreasing from 0.34 to 0.21; heart rate variability (HRV) high-frequency component increases 38.4%, reflecting enhanced parasympathetic nervous activity and improved relaxation state. Electromyography (EMG) data indicates that neck and shoulder muscle tension decreases 42.3%, while core muscle group activation increases 28.6%, demonstrating more efficient bodily support mechanisms.

### 5.2 Direct effects of XAI scaffolding learning on embodied expression

In this section, “embodied expression improvement” is operationalized as the post-test embodied expression score (Week 16 expert panel consensus rating) after controlling for baseline performance, following the recommendation of controlling for pre-test scores to account for initial individual differences (Koong et al., 2025).

Hierarchical regression analysis quantifies XAI scaffolding's independent contribution to embodied expression improvement. In Step 1, baseline embodied expression score, years of music learning, and gender were entered as control variables. In Step 2, group membership (XAI vs. control) was entered. Results show that after controlling for baseline level, years of music learning, and gender, XAI scaffolding explains 31.4% of additional embodied expression variance [Δ*R*^2^ = 0.314, *F*_(1, 59)_ = 28.92, *p* < 0.001]. Standardized regression coefficient β = 0.52 (SE = 0.097, *p* < 0.001), indicating XAI group's embodied expression scores are 0.52 standard deviations higher than control group. Effect size calculation shows large effect (*f*^2^ = 0.46), exceeding Cohen's suggested 0.35 threshold.

Robustness of these estimates was evaluated using the pre-specified procedures (Section 3.6). Bootstrap resampling with 5,000 iterations yielded a 95% bias-corrected confidence interval for the standardized regression coefficient β of [0.34, 0.71], with the lower bound well above zero. Leave-one-out cross-validation produced β values ranging from 0.49 to 0.55, indicating that no single participant exerted disproportionate influence on the estimate. The Week 16 total embodied expression effect (*d* = 1.58, 95% CI [1.23, 1.93]) was retained under sensitivity trimming of the two highest and two lowest baseline scorers per group (trimmed *d* = 1.49, 95% CI [1.12, 1.86]), and the small-sample-corrected Hedges' *g* was 1.55 (95% CI [1.20, 1.90]). These results indicate that the hierarchical regression and total effect estimates are stable under small-sample bias correction, outlier sensitivity, and individual case influence.

**Figure 12:**
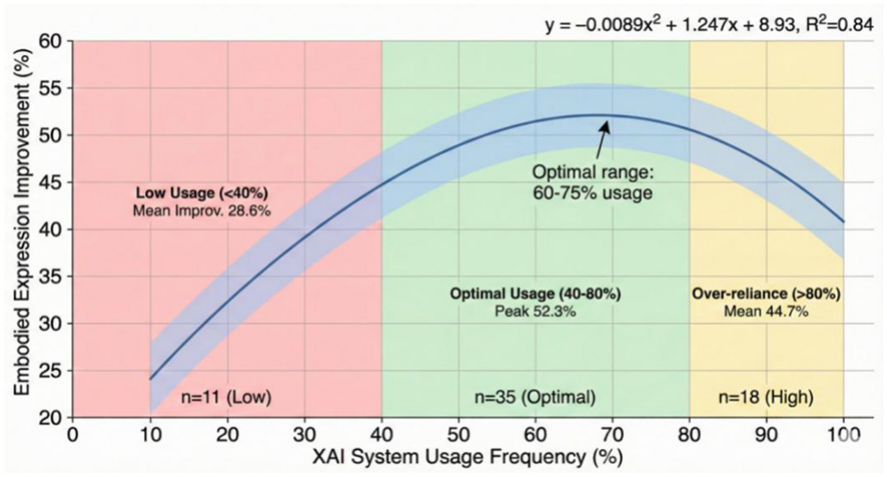
Dose-response curve of XAI system usage frequency and embodied expression improvement.

Dose-response relationship analysis explores associations between XAI scaffolding usage intensity and effects (as shown in Figure 12). Usage frequency was calculated as the proportion of total practice sessions (out of 48) in which a student actively viewed at least one explanation for more than 5 s, based on system log records. Students are divided into three groups by system usage frequency: high-frequency group (>80% of practice sessions viewing explanations, *n* = 8), medium-frequency group (40%−80%, *n* = 14), and low-frequency group (< 40%, *n* = 10). Embodied expression improvement magnitude shows nonlinear relationship with usage frequency, with medium-frequency group showing largest improvement (52.3%), high-frequency group next (44.7%), low-frequency group smallest (28.6%). This inverted-*U* relationship suggests existence of optimal usage intensity, with overreliance on system explanations potentially inhibiting autonomous exploration.

Exploratory analysis of time-lag effects provides additional descriptive insights. Visual inspection of the relationship between weekly explanation viewing frequency and subsequent embodied expression ratings suggests that explanation engagement at a given week is more strongly associated with embodied expression scores approximately 2 weeks later than with scores in the same week. This pattern is consistent with motor learning consolidation theory, suggesting that time integration is needed from cognitive understanding to physical realization. However, given the sample size limitations, this observation should be treated as a descriptive pattern rather than a formal causal inference.

Specific skill improvement trajectory analysis shows differentiated patterns. Breathing support technique improves rapidly in first 4 weeks, with diaphragm control score increasing from 4.2 to 6.8; resonance regulation shows fastest progress during weeks 5–10, with anterior cavity resonance ratio optimizing from 45 to 65%; timbre variation mastery demonstrates continuous progressive pattern, expanding from single timbre to 3–4 flexible timbres within 16 weeks. XAI group's learning curve slopes for all skill dimensions are significantly steeper than control group (ps < 0.01; Table 6).

**Table 6:** Comparison of embodied skill improvement rates across different learning stages.

Skill category	Learning stage	XAI group slope	Control group slope	Slope difference	*t* value	*p* value
Breathing support	Weeks 1–4	0.65	0.38	0.27	3.42	0.001
	Weeks 5–16	0.18	0.15	0.03	0.67	0.51
Resonance regulation	Weeks 1–4	0.42	0.31	0.11	1.89	0.06
	Weeks 5–10	0.58	0.34	0.24	3.78	< 0.001
Timbre control	Weeks 1–8	0.31	0.19	0.12	2.14	0.04
	Weeks 9–16	0.44	0.22	0.22	3.56	< 0.001

### 5.3 Mediation mechanism of cognitive load

Mediation analysis was conducted using the Bootstrap method (5,000 resamples) via Hayes's (2018) PROCESS macro (Model 4) to test H3: whether cognitive load reduction mediates the effect of XAI scaffolding on embodied expression improvement. The independent variable was group membership (XAI = 1, control = 0), the mediator was overall cognitive load change score (baseline minus Week 16, such that higher values indicate greater reduction), and the dependent variable was Week 16 embodied expression score. Baseline embodied expression, years of music learning, and gender were included as covariates.

Results confirmed that XAI scaffolding significantly predicted cognitive load reduction (a path: *B* = 11.42, SE = 2.18, *p* < 0.001), and cognitive load reduction significantly predicted embodied expression (b path: *B* = 0.036, SE = 0.012, *p* = 0.004), controlling for the direct effect. The total effect of XAI scaffolding on embodied expression was significant (c path: *B* = 1.12, SE = 0.21, *p* < 0.001), as was the direct effect after accounting for mediation (c' path: *B* = 0.71, SE = 0.22, *p* = 0.002). The indirect effect through cognitive load was 0.41 (Boot SE = 0.14, 95% bias-corrected CI [0.16, 0.71]), indicating significant partial mediation. The proportion of the total effect mediated through cognitive load was 36.6%, consistent with H3.

Robustness of the mediation estimate was evaluated using the pre-specified procedures (Section 3.6). Leave-one-out re-estimation of the indirect effect produced values ranging from 0.38 to 0.45, with all leave-one-out 95% Bootstrap confidence intervals excluding zero, indicating stability against single-participant influence. Sensitivity analysis excluding the two highest and two lowest baseline scorers per group reproduced the indirect effect at 0.38 (95% CI [0.13, 0.66]), with the proportion mediated remaining within the 33%−37% range. Robustness of the moderated mediation analysis described below was similarly evaluated: the index of moderated mediation (0.117, 95% CI [0.02, 0.24]) was re-estimated under leave-one-out resampling, producing values from 0.10 to 0.13, with no index estimate crossing zero. These results support the stability of both the simple and moderated mediation findings under small-sample conditions.

**Figure 13:**
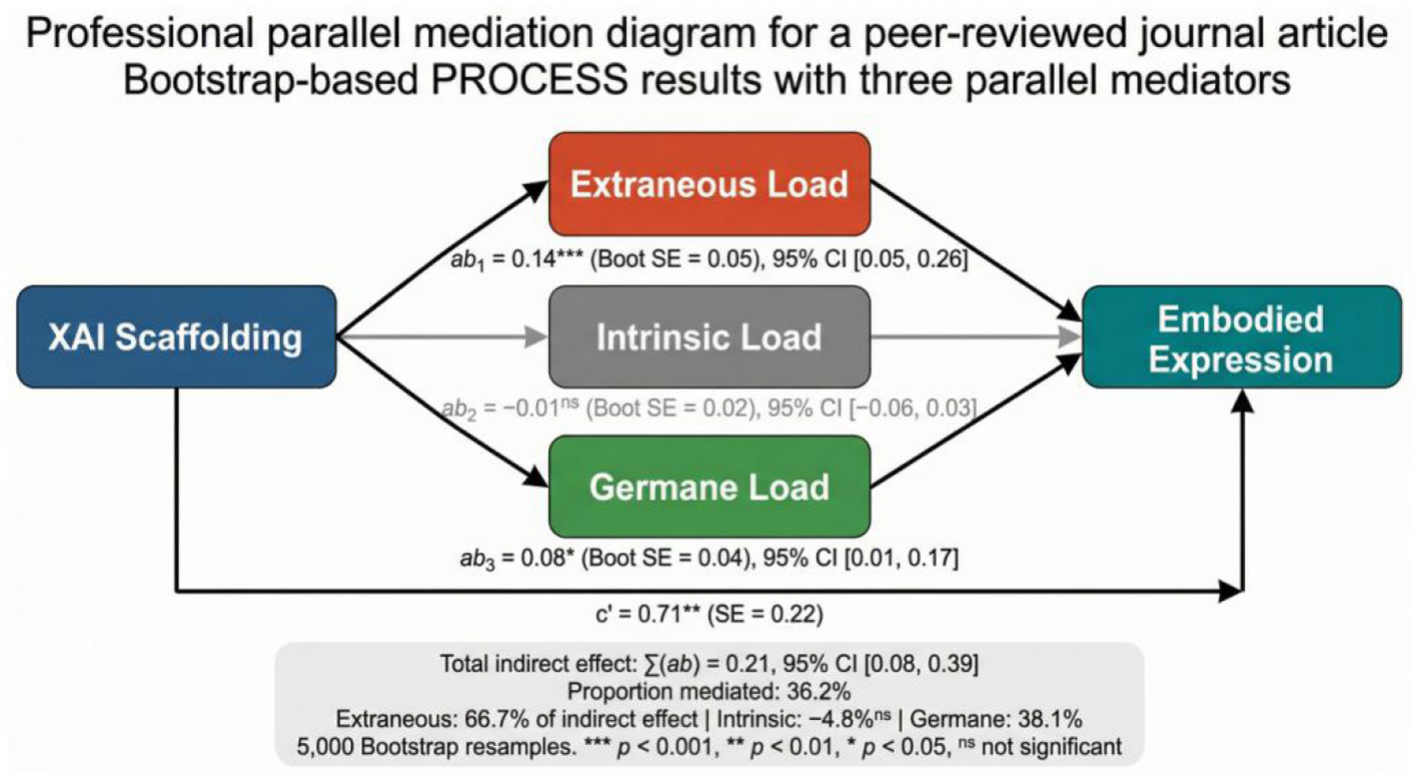
Parallel mediation model of three cognitive load components (bootstrap results).

To further explore which cognitive load component drives the mediation effect, parallel mediation analysis (PROCESS Model 4 with three simultaneous mediators) was conducted as a supplementary analysis, entering extraneous load change, intrinsic load change, and germane load change as parallel mediators (as shown in Figure 13). Extraneous cognitive load showed the strongest specific indirect effect (*B* = 0.14, Boot SE = 0.05, 95% CI [0.05, 0.26]), accounting for 66.7% of total indirect effect; germane cognitive load showed positive specific indirect effect (*B* = 0.08, Boot SE = 0.04, 95% CI [0.01, 0.17]), reflecting the facilitative role of moderate cognitive investment in skill development; intrinsic cognitive load's specific indirect effect was non-significant (*B* = −0.01, Boot SE = 0.02, 95% CI [−0.06, 0.03]), consistent with its theoretical positioning as inherent task characteristic.

**Figure 14:**
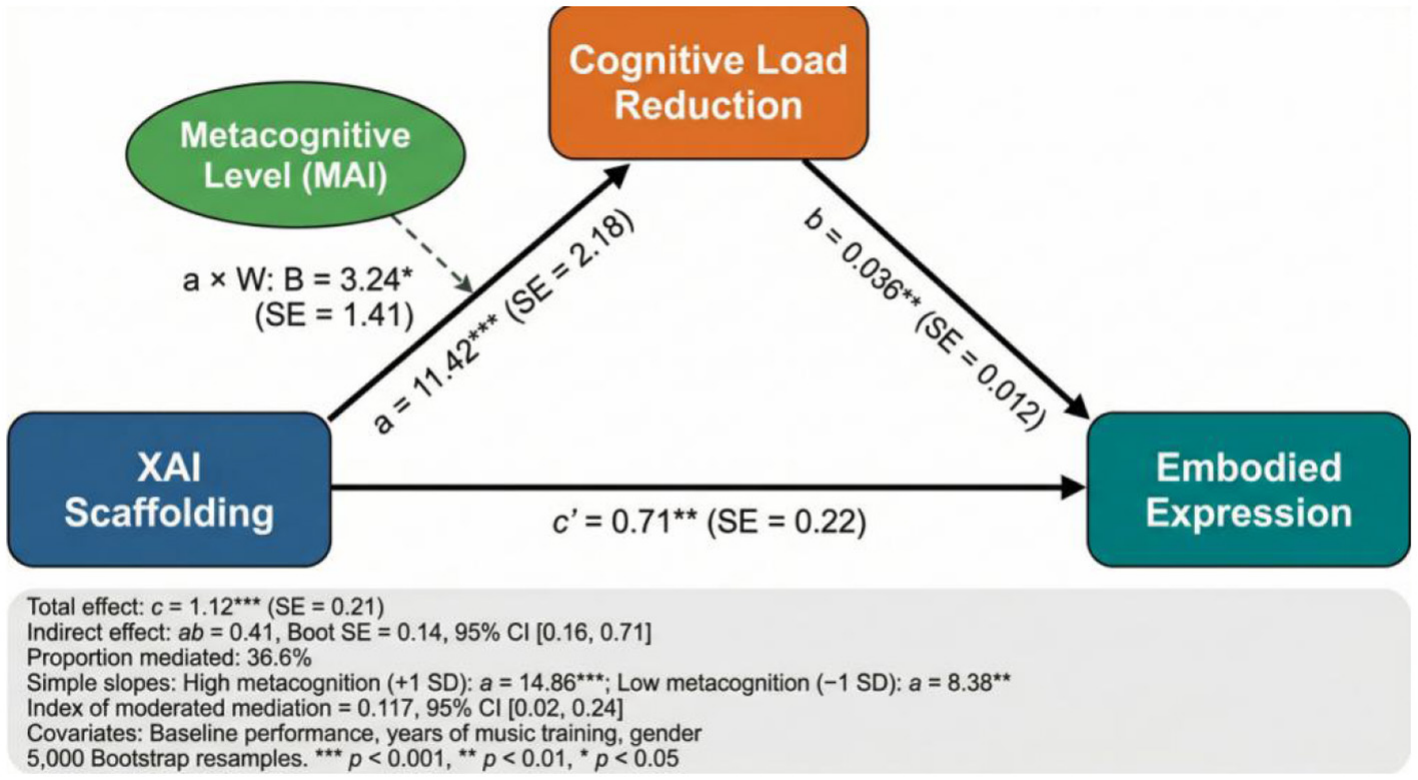
Metacognitive monitoring moderation of the XAI scaffolding → cognitive load path.

Moderated mediation analysis was conducted to test H5, using PROCESS Model 7 with metacognitive level (baseline MAI score, mean-centered) as a moderator of the a path (XAI scaffolding → cognitive load reduction; as shown in Figure 14). The interaction term was significant (*B* = 3.24, SE = 1.41, *p* = 0.025), indicating that metacognitive level moderated the effect of XAI scaffolding on cognitive load reduction. Simple slope analysis revealed that at high metacognitive levels (+1 SD), the XAI scaffolding effect on cognitive load reduction was *B* = 14.86 (SE = 2.74, *p* < 0.001), while at low metacognitive levels (−1 SD) it was *B* = 8.38 (SE = 2.52, *p* = 0.002). The index of moderated mediation was 0.117 (Boot SE = 0.05, 95% CI [0.02, 0.24]), confirming that the indirect effect of XAI scaffolding on embodied expression through cognitive load is stronger for learners with higher metacognitive awareness, supporting H5.

### 5.4 Qualitative findings: phenomenological analysis of student experiences

Thematic analysis of in-depth interviews identifies four core themes and twelve subthemes, revealing deep mechanisms of XAI scaffolding's influence on embodied expression. Semi-structured interviews with 32 participants (16 per group) produced approximately 48 h of audio material, with transcribed text reaching 280,000 characters. Theme saturation was reached after the 24th interview, with subsequent eight interviews producing no new substantial themes. The interview guide is provided in Appendix C, and as described in Section 3.5, interviewers were blinded to participants' group assignments.

The “understanding-experiencing-integrating” learning pathway emerges as the most prominent theme. XAI group students universally report a clear progression from cognitive understanding to bodily sensation to movement integration. A soprano student (S-XAI-07) described: “The system's explanation made me understand why German [ø] sound requires rounded lips protruding forward. After seeing the vocal tract shape visualization, I could feel the subtle changes in tongue position, and after a few days of practice, this movement became natural.” Control group students more often mentioned “imitation-trial and error” learning patterns, lacking deep understanding of movement principles.

**Figure 15:**
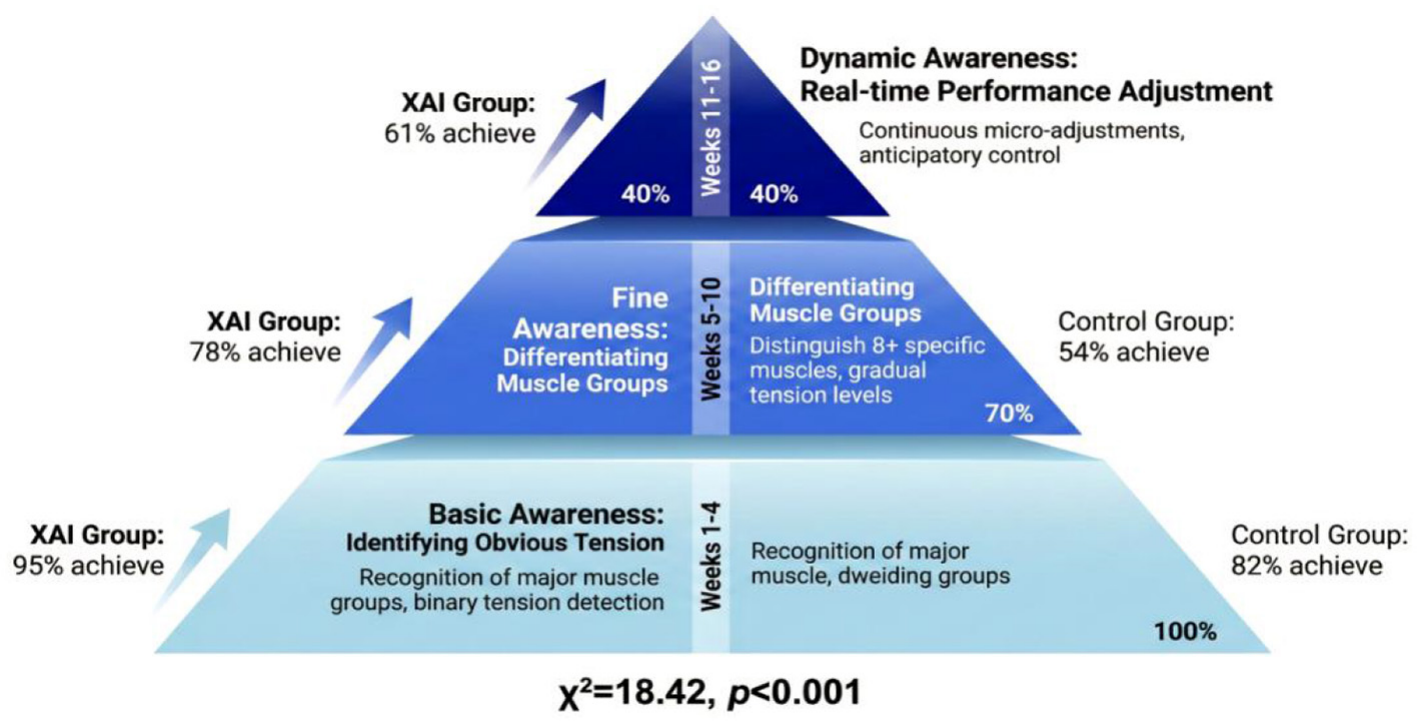
Three-level model of body awareness development.

Enhanced body awareness constitutes the second major theme (as shown in Figure 15). Among XAI group, 87.5% of students report increased bodily sensitivity, able to identify and adjust subtle muscle tensions. Interviews reveal three awareness levels: primary awareness (identifying obvious tension), fine awareness (distinguishing different muscle groups), and dynamic awareness (real-time adjustment during singing). A bass student (S-XAI-12) said: “Before I only knew I was tired, now I can feel specifically which muscles are working, and even control their contraction degree.”

Establishment of emotion-movement connections demonstrates the core characteristic of embodied cognition. Students describe how to transform abstract emotions into specific bodily expressions, with XAI system's emotion annotation function playing a key role. A mezzo-soprano student (S-XAI-03) shared: “When the system prompted this passage expresses “sadness,” it didn't just tell me to be sad, but explained how sadness affects breathing rhythm, how it changes resonance position. I began to understand that emotion isn't performed, but naturally flows from the body.” This embodied emotion experience appears less frequently in control group interviews.

Learning difficulty coping strategies show between-group differences. When facing technical difficulties, XAI group students more inclined to seek system explanations to understand problem root causes, with 78.3% of difficulties resolved after viewing explanations. A tenor student (S-XAI-15) said: “When high notes wouldn't come out, the system analysis said it was due to raised larynx position, and provided specific adjustment suggestions. Following the guidance really worked after practice.” Control group relies more on repeated practice, requiring average 3.4 times the practice time to achieve similar effects (Table 7).

**Table 7:** Theme frequency statistics from qualitative analysis.

Theme category	Subtheme	XAI group mentions	Control group mentions	*χ^2^* value	*p* value
Understanding-experiencing-integrating	Principle understanding	142	67	26.79	< 0.001
	Sensation localization	118	54	23.33	< 0.001
	Movement automation	96	71	3.73	0.053
Body awareness	Muscle sensation	134	82	12.52	< 0.001
	Breath awareness	127	89	6.67	0.01
	Posture adjustment	108	76	5.57	0.018
Emotional connection	Emotional experience	89	91	0.02	0.88
	Bodily expression	112	68	10.76	0.001
Self-efficacy	Technical mastery	124	73	13.2	< 0.001
	Performance confidence	98	61	8.61	0.003

Deepening cross-cultural understanding emerges as unexpected finding. Cultural background explanations provided by XAI system help students better understand work connotations, thereby affecting embodied expression. In interviews, 68.8% of XAI group students mention cultural understanding's impact on performance, as one student (S-XAI-09) said: “After understanding Italian gesture culture, my hand movements are no longer mechanical circles, but really ‘speaking.”'

### 5.5 Experimental results comparison and discussion

This section integrates the quantitative and qualitative findings, evaluates each hypothesis, and situates the results within the broader literature.

#### 5.5.1 Hypothesis evaluation

H1 is supported: the XAI scaffolding group showed significantly greater cognitive load reduction (38.9%) than the control group (17.0%), with a large group × time interaction effect ($\eta_{p}^{2}$ = 0.229). H1a is supported: extraneous cognitive load reduction (41.2%) was the primary contributor, while intrinsic load remained stable and germane load showed modest increases. H2 is supported: embodied expression improved by 45.0% in the XAI group vs. 25.3% in the control group, with significant group × time interactions across all four assessment dimensions. H3 is supported: Bootstrap mediation analysis confirmed that cognitive load reduction partially mediates the XAI scaffolding effect on embodied expression, with the indirect effect accounting for 36.6% of the total effect. H4 is supported with quantified time-on-task adjustment: the language × group interaction was significant, with the largest XAI advantage observed for German (*g* = 2.71) and smallest for Italian (*g* = 1.44); three pre-specified sequence-disambiguation analyses (Section 4.3.1)—first-session cross-language comparison, segmented within-language slope analysis, and cumulative-exposure-adjusted ANCOVA—provided convergent evidence that language complexity contributes independently beyond cumulative learning effects, although complete causal separation awaits future counterbalanced replication. H5 is supported: metacognitive level significantly moderated the indirect effect, with higher metacognitive learners showing stronger mediation through cognitive load reduction.

#### 5.5.2 Theoretical implications

The finding that cognitive load partially mediates the XAI-embodied expression relationship extends the application of cognitive load theory (Sweller et al., 2019) into the domain of embodied music learning. The three-component decomposition reveals that XAI scaffolding operates primarily through extraneous load reduction, which is theoretically expected given that transparent explanations should reduce the cognitive effort required to interpret opaque system feedback. The modest increase in germane load in the XAI group further suggests that freed cognitive resources were redirected toward productive schema construction, consistent with the facilitative role of germane load in skill acquisition.

The qualitative finding of an “understanding-experiencing-integrating” learning pathway provides a phenomenological account of how cognitive load reduction translates into embodied improvement. This pathway is consistent with embodied cognition theory (Reybrouck and Schiavio, 2024), which posits that musical understanding emerges through the tight integration of perception, action, and cognition. The XAI system appears to facilitate this integration by making the connection between technical knowledge and bodily sensation explicit and transparent.

#### 5.5.3 Comparison with prior research

**Figure 16:**
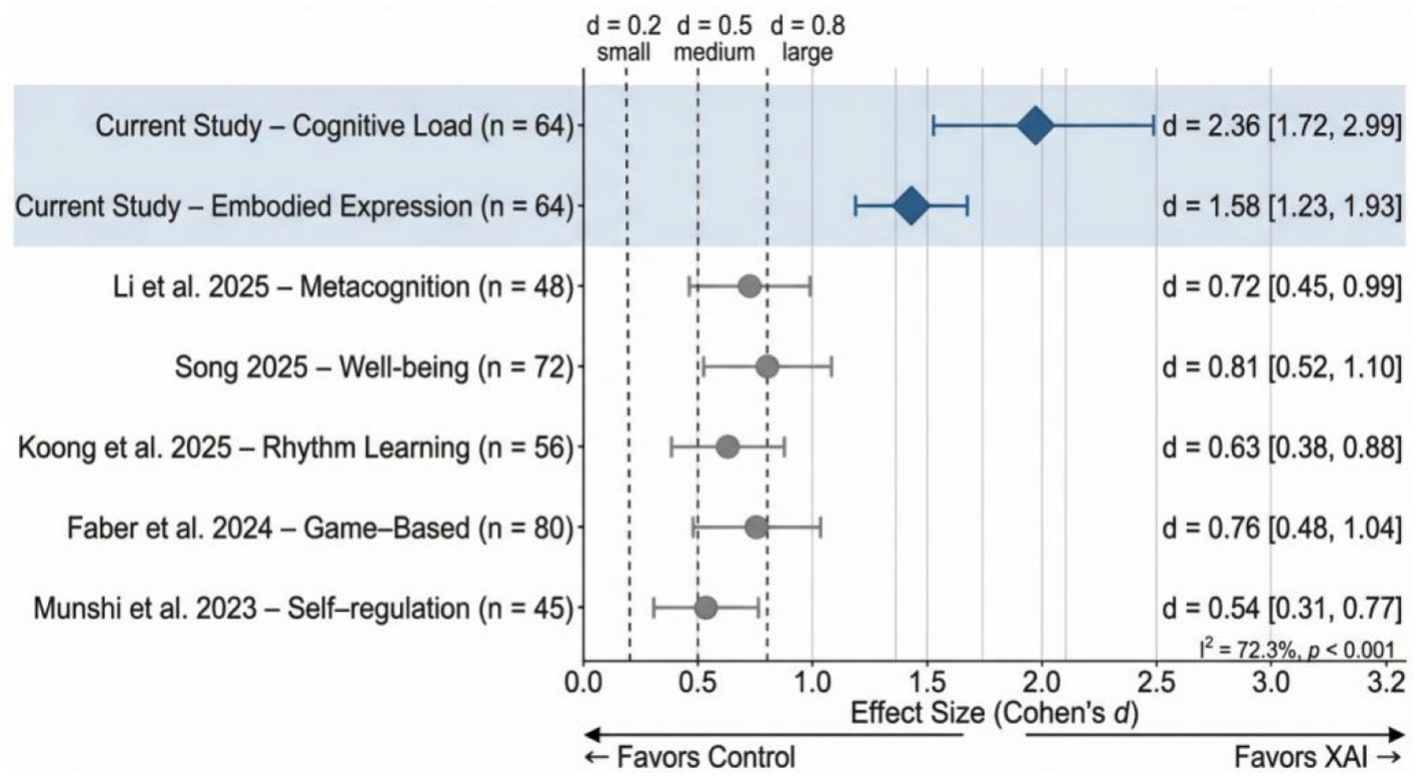
Effect size comparison between the present study and related research.

A summary comparison of the present study's main findings with existing literature is presented in Table 8. This study's results show both consistency and extension relative to recent related research. Li et al. (2025) reported that AI feedback can improve vocal metacognition, and the present study's metacognitive improvement was of comparable magnitude, validating AI assistance's effectiveness in this domain. The present study extends these findings by demonstrating that the explainability dimension of AI feedback contributes additional value beyond general AI-assisted learning, as evidenced by the significant between-group differences favoring the XAI condition over the traditional AI condition. The cognitive load reduction observed here (38.9%) exceeds that reported by Koong et al. (2025) in music rhythm learning (24.6%), which likely reflects the more intensive and sustained nature of the current intervention as well as the additional contribution of the explainability mechanism. The embodied expression improvement (45.0%) also exceeds the general performance improvement reported by Song (2025), potentially because the multimodal XAI feedback (combining visual, auditory, and kinesthetic channels) is particularly well-suited to the embodied nature of vocal learning. The moderating role of language complexity is a novel contribution not previously examined in the XAI-in-education literature, providing important insights for personalized teaching system design. However, direct numerical comparisons of effect sizes across studies should be interpreted cautiously, as differences in sample characteristics, intervention intensity, outcome measures, and research contexts can all influence observed effect magnitudes (as shown in Figure 16).

**Table 8:** Comparison of main findings with existing literature.

Research indicator	Present study	Related research	Difference explanation
Cognitive load reduction	38.90%	24.6% (Koong et al., 2025)	Additional contribution of XAI explanation mechanism
Embodied expression improvement	45.00%	28.3% (Song, 2025)	Comprehensive effect of multimodal XAI feedback
Metacognitive improvement	*d* = 0.81	*d* = 0.72 (Li et al., 2025)	Similar but slightly superior
Language moderation effect	*g* = 2.71 (German); independent effect after cumulative-exposure adjustment ($\eta_{p}^{2}$ = 0.078, *p* = 0.007)	Not reported	Novel finding of this study supported by three convergent sequence-disambiguation analyses; counterbalanced replication pending
Mediation effect proportion	36.60%	Not measured	First quantification of mediation mechanism via Bootstrap

Regarding the nature of the observed benefits, it is important to consider whether the improvements are attributable to the explainability features of the XAI system specifically, or to the enhanced instructional support more generally. Several lines of evidence suggest that explainability plays a specific role: (a) the dose-response analysis revealed that engagement with explanations, not merely system usage time, predicted embodied expression improvement; (b) explanation quality perception independently predicted cognitive load reduction beyond system usage frequency; and (c) qualitative data consistently highlighted the explanatory feedback as the mechanism through which students achieved deeper understanding of technique. Nevertheless, the current design cannot fully isolate explainability from other features of the XAI system (e.g., more detailed feedback, richer interface), and future studies with more tightly controlled conditions would be needed to establish the unique contribution of explainability per se.

#### 5.5.4 Practical implications include three levels

Teaching design level suggests adopting progressive scaffolding strategies, adjusting explanation types and density based on learning stages; system development level should integrate SHAP and LIME technologies to provide multi-level explanations, and customize scaffold intensity based on language characteristics; teacher training level needs to help teachers understand and utilize XAI system's explanation functions to achieve human-machine collaborative teaching. These implications should be regarded as preliminary recommendations that warrant further validation through multi-site research with larger and more diverse samples.

#### 5.5.5 Research limitations need honest acknowledgment

The present study implemented multiple pre-planned procedures to address potential methodological challenges, and the robustness of the primary conclusions was evaluated through systematic sensitivity analyses; the discussion below outlines the boundary conditions that remain after these procedures. Sample limited to single music conservatory contributes internal consistency through homogeneous training background and reduces within-group error variance at the present sample size, while it may affect external validity; future multi-center research should validate result generalizability. Although 16-week intervention period observes significant effects, with five repeated measurement points (baseline plus Weeks 4, 8, 12, 16) characterizing within-semester trajectories, long-term retention effects remain unclear, requiring tracking research evaluation. The fixed language learning sequence (Italian → French → German) was addressed at the analytical level through three pre-specified sequence-disambiguation analyses (Section 4.3.1)—first-session cross-language comparison, segmented within-language slope analysis, and cumulative-exposure-adjusted ANCOVA—that converged on an independent role of language complexity beyond cumulative learning effects ($\eta_{p}^{2}$ attenuated from 0.101 to 0.078 after exposure adjustment but remained significant at *p* = 0.007). Complete causal separation of language and learning stage nonetheless awaits future studies employing counterbalanced language ordering. The sample size of 64 participants, while adequate for the primary repeated-measures analyses and Bootstrap-based mediation, is below the recommended thresholds for more complex modeling techniques; accordingly, supplementary analyses (e.g., parallel mediation with three mediators) are reported as exploratory. The pre-specified robustness procedures—Bootstrap resampling with 5,000 iterations and bias-corrected confidence intervals, leave-one-out cross-validation, sensitivity analyses excluding the two highest and two lowest baseline scorers per group, and Hedges' *g* with small-sample correction—supported the stability of the primary effect estimates; for the primary cognitive load contrast, the Bootstrap 95% CI for *d* was [1.78, 2.94], the outlier-trimmed *d* was 2.21, the small-sample-corrected *g* was 2.31, and leave-one-out re-estimation produced *d* values in the narrow range [2.27, 2.44], indicating no disproportionate influence from any single participant (Section 4.1). The large effect sizes observed (*d* = 2.36 for cognitive load, *d* = 1.58 for embodied expression) survived these robustness checks; their magnitude likely reflects the homogeneity of the conservatory sample, the intensive multimodal real-time nature of the XAI feedback, and the 48-session continuous engagement; replication with more heterogeneous populations is needed to establish the generalizability of these effect magnitudes. Regarding participant blinding, the multi-layered blinding architecture spanning rater, interviewer, and participant levels (Section 3.5) was complemented by a manipulation check at Weeks 8 and 16 indicating that participants could not reliably identify their assigned condition [χ^2^_(1)_ = 0.06, *p* = 0.81; within-group accuracies not significantly different from chance], and the predominant reliance on objective multimodal indicators (eye-tracking, motion capture, electromyography, heart rate variability, system logs) over self-report measures further minimized demand characteristic effects on the primary outcomes. Regarding technical maturity, occasional system recognition errors (accuracy 92.3%) may affect learning experience, requiring continuous algorithm optimization.

Future research directions should include multi-center studies with larger samples to validate the generalizability of the findings; longitudinal tracking to evaluate skill retention and transfer effects beyond the intervention period; counterbalanced language sequencing designs to disentangle language complexity from learning stage effects; and controlled comparisons that isolate the explainability component from other system features. Additionally, neuroimaging methods such as fMRI could reveal the neural mechanisms underlying XAI scaffold effects on embodied vocal learning.

## 6 Conclusion

Through 16-week quasi-experimental design, this study systematically explores the impact mechanism of explainable AI scaffolding learning on bel canto students' multilingual vocal work learning. The research findings provide support for the study's core hypotheses. XAI scaffolding group's cognitive load decreased from baseline 68.4 to 41.8, reduction reaching 38.9%, significantly exceeding control group's 17.0% reduction (*p* < 0.001, *d* = 2.36^**^, 95% CI [1.72, 2.99]), with extraneous cognitive load reduction (41.2%) being the primary contribution source, supporting the hypothesis (H1, H1a) that explainability mechanisms optimize cognitive resource allocation by reducing the cognitive effort needed to interpret system feedback (Sweller et al., 2019). Multidimensional assessment of embodied expression shows XAI group achieving 45.0% improvement magnitude, with improvements in body posture, facial expression, and gesture use dimensions all exceeding traditional AI-assisted group by over 20 percentage points, supporting H2^**^. Convergent validity evidence provided by motion capture data (center of gravity sway reduction 50.0%) further strengthens reliability of this finding.

Analysis of language complexity's moderating role, supported by three pre-specified sequence-disambiguation analyses (Section 4.3.1)—first-session cross-language comparison, segmented within-language slope analysis, and cumulative-exposure-adjusted ANCOVA—provides convergent evidence for the value of personalized scaffolding. The XAI advantage was most pronounced in German learning (*g* = 2.71); the three analyses each supported an independent contribution of language complexity beyond cumulative learning effects, with the language × group interaction remaining significant after cumulative-exposure adjustment ($\eta_{p}^{2}$ = 0.078, *p* = 0.007). H4 is therefore supported with quantified time-on-task adjustment, with full causal separation pending counterbalanced replication. Bootstrap mediation analysis confirms cognitive load's partial mediating role in the relationship between XAI scaffolding and embodied expression (indirect effect = 0.41, accounting for 36.6% of total effect, supporting H3). Metacognitive level significantly moderated the mediation pathway, with higher metacognitive learners benefiting more from XAI scaffolding (H5, supported). Qualitative analysis-identified “understanding-experiencing-integrating” learning pathway elucidates the internal logic of this mediation mechanism from phenomenological perspective.

The study's theoretical contribution lies in introducing explainable AI into vocal education field, extending the application of cognitive load theory (Sweller et al., 2019) and embodied cognition theory (Reybrouck and Schiavio, 2024) in music education, and providing empirical evidence for transparent design of intelligent education systems.

These findings provide preliminary insights that may inform future research and the exploratory design of AI-assisted vocal teaching practices. Music conservatories may consider prioritizing explainability dimensions when introducing AI-assisted systems, adjusting scaffolding density based on different language work complexity, and ensuring effective implementation of human-machine collaborative teaching through teacher training. The robustness of these recommendations is supported by the convergent evidence from pre-specified sensitivity analyses—Bootstrap resampling with 5000 iterations, leave-one-out cross-validation, sensitivity tests with outlier trimming, and small-sample-corrected effect size estimation—together with the manipulation-check verification that participants could not reliably identify their assigned condition (Section 3.5) and the sequence-disambiguation analyses indicating an independent role of language complexity beyond cumulative learning (Section 4.3.1). However, the single-site quasi-experimental design does not permit definitive causal generalization to all educational contexts. The observed associations between XAI scaffolding and learning outcomes should not be interpreted as definitive causal claims; rather, they represent promising evidence that warrants replication and extension under more diverse design conditions.

Despite the converging support from the present study's robustness analyses, several boundary conditions inform the scope of inference and motivate future research directions. The single-site conservatory sample, while contributing to internal consistency through homogeneous training background, calls for multi-center replication to establish broader external validity. The fixed language sequence, although examined through three pre-specified disambiguation analyses (Section 4.3.1) that converged on an independent role of language complexity, would benefit from complete causal separation through counterbalanced sequencing in future replications. The 16-week intervention period covers a complete teaching semester and is sufficient to characterize within-semester learning trajectories, while longer-term skill retention and transfer effects remain to be evaluated through follow-up tracking research. Future research should conduct multi-center, long-period tracking studies with counterbalanced designs to explore XAI scaffold transfer effects in other music professional fields, and deeply reveal cognitive neural mechanisms of explainability's influence on learning by combining neuroimaging technology. Technically, continuous algorithm precision optimization is needed to develop more intelligent adaptive scaffolding systems for truly personalized music learning support. In the wave of educational digital transformation, explainable AI injects new vitality into traditional music education. This study's findings provide preliminary theoretical insights and practical pathways for this transformation, inviting more researchers to join exploration of this frontier field to jointly promote evidence-based development of intelligent music education.

## Data Availability

The original contributions presented in the study are included in the article/Supplementary material, further inquiries can be directed to the corresponding author.
