# Supplementary material for: Can explainable AI scaffolding reduce cognitive load and enhance embodied expression? A quasi-experimental study of multilingual vocal learning in Bel Canto education
Source: Front Psychol. 2026 Jun 23;17:1835027. doi: 10.3389/fpsyg.2026.1835027 (PMC13337817; doi:10.3389/fpsyg.2026.1835027)
Supplement: Supplementary file 1 [file Supplementary_File_1.docx]

# **Appendix A: Core Mathematical Formulations of XAI Explanation Mechanisms**

This appendix provides the core mathematical formulations underlying the three explanation mechanisms implemented in the XAI scaffolding learning system described in Section 3.3. The formulations include the deep learning model architecture (A.1), SHAP value computation for global explanation (A.2), LIME local approximation for local explanation (A.3), counterfactual distance minimization (A.4), and scaffold intensity adaptive adjustment (A.5).

## **A.1 Deep Learning Model Architecture**

The timbre analysis module employs Mel-spectrogram feature extraction combined with convolutional neural networks. The network structure with *n* = 5 layers is formulated as:

$\text{f}\left( \text{x} \right)\text{=}\text{σ}\left( \text{W}_{\text{n}}\text{⋅⋯}\text{σ}\left( \text{W}_{\text{2}}\text{⋅}\text{σ}\left( \text{W}_{\text{1}}\text{⋅}\text{x}\text{+}\text{b}_{\text{1}} \right)\text{+}\text{b}_{\text{2}} \right)\text{⋯+}\text{b}_{\text{n}} \right)$ (A1)

where *x* represents the input audio feature vector (128 Mel bands × variable time frames extracted from 48kHz/24-bit audio recordings), *W*ᵢ and *b*ᵢ represent the weight matrix and bias vector of layer *i*, and σ is the ReLU activation function.

## **A.2 SHAP Value Computation for Global Explanation**

Global explanation calculates each feature's contribution to overall scoring through SHAP (SHapley Additive exPlanations) values. The SHAP value for feature *i* is computed as:

$\text{φ}_{\text{i}}\text{=}\sum_{\text{S}\text{⊆}\text{F}\text{\textbackslash\{}\text{i}\text{\}}} \left[ \frac{\text{|}\text{S}\text{|!}\left( \text{|}\text{F}\text{|−|}\text{S}\text{|−1} \right)\text{!}}{\text{|}\text{F}\text{|!}} \right]\text{×}\left[ \text{f}_{\text{S}\text{∪\{}\text{i}\text{\}}}\left( \text{x}_{\text{S}\text{∪\{}\text{i}\text{\}}} \right)\text{−}\text{f}_{\text{S}}\left( \text{x}_{\text{S}} \right) \right]$ (A2)

where φᵢ represents the SHAP value of feature *i*, *F* is the set of all features (pronunciation accuracy, pitch precision, rhythmic alignment, tonal quality, and expressive interpretation), *S* is a feature subset, and $\text{f}_{\text{S}}$ is the model trained on feature subset *S*. The summation iterates over all possible subsets of features excluding feature *i*, weighted by the combinatorial coefficient that ensures fair attribution across all possible orderings. The resulting SHAP feature contribution bar chart (displayed in the learner-facing interface as described in Section 3.3) allows learners to see which performance dimensions most strongly influenced their overall score.

## **A.3 LIME Local Approximation for Local Explanation**

Local explanation uses LIME (Local Interpretable Model-agnostic Explanations) to generate linear approximation models for specific predictions. The objective function is:

$\text{ξ}\left( \text{x} \right)\text{=}\text{argmin}_{\text{g}\text{∈}\text{G}}\text{ }\text{L}\left( \text{f}\text{, }\text{g}\text{, }\text{π}_{\text{x}} \right)\text{+Ω}\left( \text{g} \right)$ (A3)

where *f* is the original deep learning model, *g* is an interpretable model (linear model in this implementation), *G* is the class of interpretable models, *L* is a loss function measuring how unfaithful *g* is in approximating *f* in the locality defined by π_x, and Ω(*g*) is a complexity measure of the interpretable model. The locality kernel π_x is defined as:

$\text{π}_{\text{x}}\left( \text{z} \right)\text{=}\text{exp}\left( \text{−}\frac{\text{d}\left( \text{x}\text{, }\text{z} \right)^{\text{2}}}{\text{σ}^{\text{2}}} \right)$ (A4)

where *d*(*x*, *z*) is a distance function and σ is a width parameter. In the system implementation, LIME generates phrase-level explanations by perturbing input features around a specific vocal phrase and observing how the model's score changes, producing a linear approximation indicating which features (e.g., vowel openness, consonant articulation, breath support) most influenced the score for that particular phrase.

## **A.4 Counterfactual Distance Minimization**

Counterfactual explanation finds the minimal changes needed to alter the system's evaluation by solving:

$\text{x}^{\text{∗}}\text{=}\text{argmin}_{\text{x}\text{′}}\text{ }\text{d}\left( \text{x}\text{, }\text{x}\text{′} \right)\text{ }\text{s}\text{.}\text{t}\text{. }\text{f}\left( \text{x}\text{′} \right)\text{≠}\text{f}\left( \text{x} \right)$ (A5)

where *d*(*x*, *x*') is a distance measure between the original input *x* and the counterfactual instance *x*', using weighted Euclidean distance to account for feature importance differences:

$\text{d}\left( \text{x}\text{, }\text{x}\text{′} \right)\text{=}\sqrt{\sum_{\text{i}} \text{w}_{\text{i}}\left( \text{x}_{\text{i}}\text{−}{\text{x}\text{′}}_{\text{i}} \right)^{\text{2}}}$ (A6)

where *w*ᵢ represents the importance weight of feature *i*, derived from the global SHAP values (Equation A2). This weighting ensures that counterfactual explanations prioritize changes to the most impactful performance dimensions. In practice, the counterfactual engine answers questions such as "What would I need to change to improve my score on this passage?" by identifying the smallest set of adjustments that would move the learner's performance into a higher scoring category.

## **A.5 Scaffold Intensity Adaptive Adjustment**

The system's scaffold intensity is adaptively adjusted based on a sliding window assessment of learner performance, using an exponential weighted moving average (EWMA) algorithm:

$\text{P}_{\text{t}}\text{=}\text{α}\text{S}_{\text{t}}\text{+}\left( \text{1−}\text{α} \right)\text{P}_{\text{t}\text{−1}}$ (A7)

where *P*ₜ is the scaffold intensity parameter at time *t*, *S*ₜ is the current performance score, and α = 0.3 is the smoothing coefficient. This coefficient was selected through pilot testing to balance responsiveness to performance changes with stability of scaffold adjustment. The scaffold intensity parameter *P*ₜ determines the level of detail in explanations, the frequency of proactive feedback, and the degree of autonomy granted to the learner, following the progressive withdrawal principles described by Wood et al. (1976).

# **Appendix B: Teacher Dashboard and SHAP Feature Contribution Maps**

**
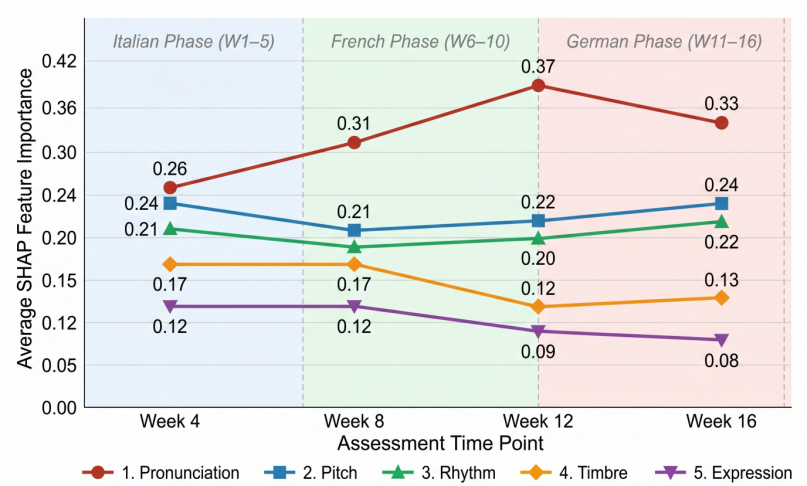
**

## **B.1 Teacher Dashboard - Class-Level SHAP Feature Importance Trends**

**
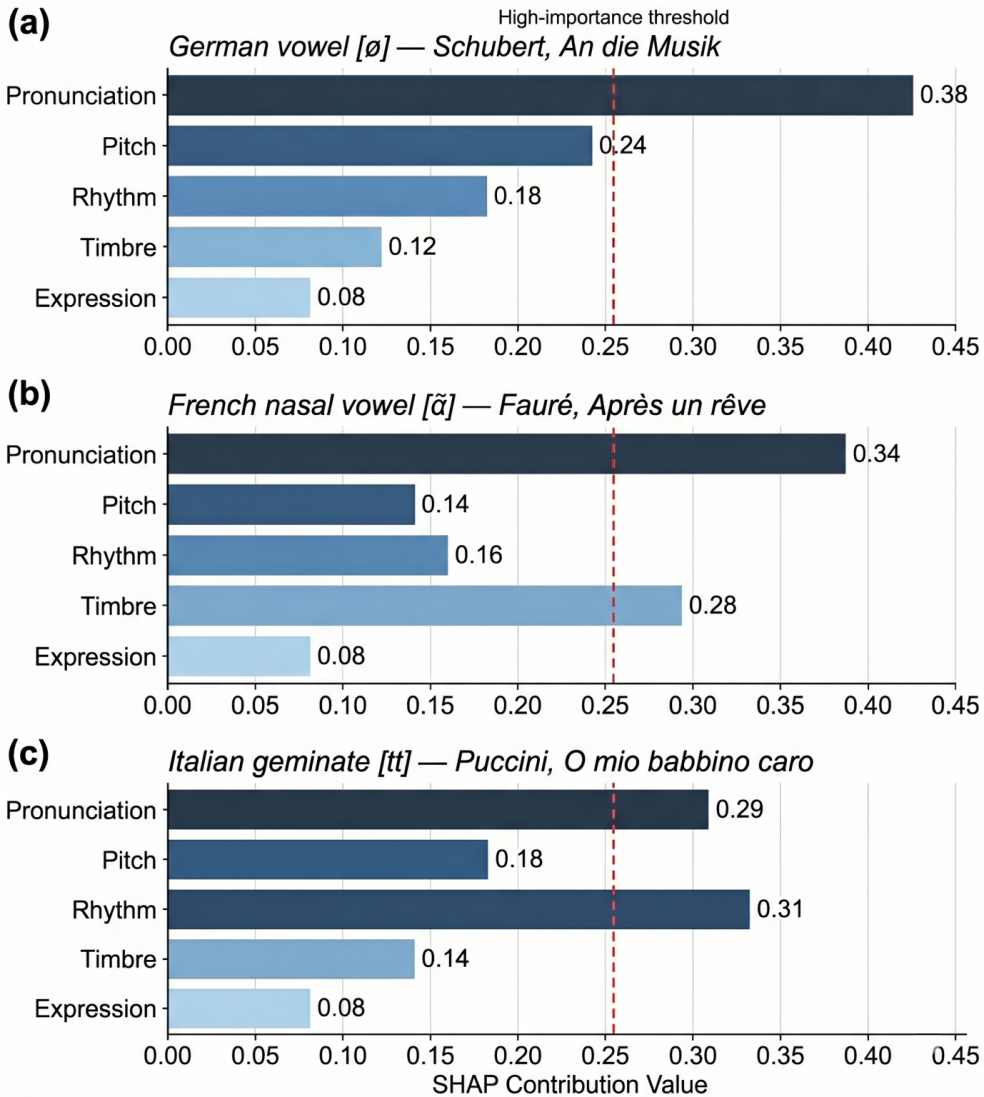
**

## **B.2 SHAP Feature Contribution Maps for Three Pronunciation Errors**

**
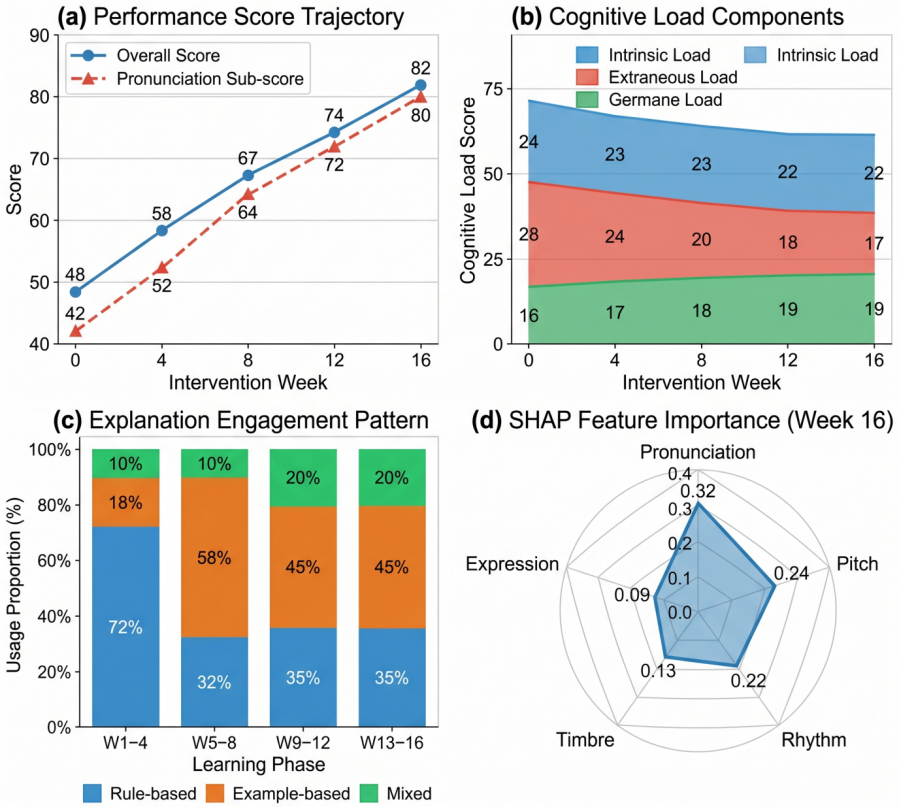
**

## **B.3 Individual Student Progress Dashboard Panel**

# **Appendix C: Semi-Structured Interview Guide**

The following interview guide was used for the semi-structured interviews conducted during the post-test phase (Weeks 17–18). A total of 32 participants (16 from the XAI scaffolding group and 16 from the control group) were purposively selected to represent diverse voice parts, performance levels, and system usage patterns. Each interview lasted approximately 45–90 minutes and was audio-recorded with participant consent (Section 3.5). Interviewers were trained research assistants who were not informed of participants' group assignments, maintaining interviewer blinding to reduce potential bias. The guide was piloted with 4 non-sample participants and refined based on pilot feedback.

Note: Questions marked with **[XAI only]** were asked only to participants in the XAI scaffolding group. Questions marked with **[Control only]** were asked only to participants in the control group. All other questions were asked to both groups. Follow-up probes (indicated by sub-questions) were used flexibly based on participant responses.

## **C.1 Topic 1: Overall Learning Experience and Perceived Progress**

1. Can you describe your overall experience of learning multilingual vocal works over the past 16 weeks? What were the highlights and low points?

2. Compared to the beginning of the semester, how do you feel your vocal technique has changed? In what specific areas do you feel you have improved the most?

3. Of the three languages you studied (Italian, French, German), which did you find most challenging? Why? How did your approach to learning differ across languages?

4. Did you notice any differences in how quickly you progressed with each language? If so, what do you think contributed to these differences?

5. Looking back at the learning sequence (Italian → French → German), did you feel that earlier language learning helped you with later languages? In what ways?

## **C.2 Topic 2: Interaction with the AI System and Understanding of Feedback**

6. How would you describe your experience using the AI-assisted practice system? Was it helpful? In what ways?

**[XAI only]** 7. The system provided explanations of its feedback (e.g., why you received a certain score, what specific changes could improve your performance). How useful were these explanations to you?

a. Can you give a specific example of an explanation that was particularly helpful?

b. Were there times when the explanations were confusing or unhelpful? Please describe.

c. Did you prefer the rule-based explanations (textual descriptions of phonetic rules) or the example-based explanations (visual/audio comparisons with reference recordings)? Did this preference change over time?

d. How did you typically use the SHAP feature contribution chart? Did it help you prioritize what to work on?

**[XAI only]** 8. Did the explanations change how you practiced? Can you describe how you used the system's feedback during a typical practice session?

**[Control only]** 9. During practice sessions, when the system indicated an error, how did you usually try to correct it? What strategies did you use to understand what went wrong?

**[Control only]** 10. Were there times when you wished the system had provided more detailed feedback about why you received a particular score? What additional information would have been helpful?

11. How much did you trust the system's feedback? Did your level of trust change over the 16 weeks? What influenced your trust?

## **C.3 Topic 3: Changes in Body Awareness and Expressive Performance**

12. Over the past 16 weeks, have you noticed any changes in your awareness of your body during singing? Please describe these changes in as much detail as possible.

a. Are you more aware of specific muscle groups (e.g., diaphragm, larynx, tongue)? Which ones?

b. Has your breathing pattern changed? How?

c. Do you feel your posture during singing has changed? In what ways?

13. Can you describe the process by which your understanding of a vocal technique translated into actual physical changes in your singing? *[Probe for understanding → experiencing → integrating progression]*

**[XAI only]** 14. Did the system's feedback help you connect your physical sensations with your vocal production? Can you describe a specific moment when this connection became clear?

15. How has your ability to express emotions through singing changed? Can you provide a specific example of a passage or song where your expressive performance improved?

a. Do you feel that emotions are something you "perform" externally, or something that naturally flows from your body? Has this perception changed?

16. Do you feel that your gestures and body movements during performance have become more natural or coordinated? What contributed to this change?

17. Has your comfort level and stage presence when performing in front of others changed? What aspects of your training contributed to these changes?

## **C.4 Topic 4: Challenges Encountered and Coping Strategies**

18. What was the most significant challenge you faced during this learning period? How did you deal with it?

19. Were there moments when you felt overwhelmed by the amount of information you needed to process (e.g., pronunciation, melody, expression simultaneously)? How did you manage this cognitive demand?

a. **[XAI only]** Did the system's explanations help reduce this feeling of being overwhelmed? In what way?

b. **[Control only]** What strategies did you develop on your own to manage the complexity of multilingual learning?

20. When you encountered a particularly difficult passage, what was your typical approach to mastering it? Did this approach change over the 16 weeks?

**[XAI only]** 21. Were there any aspects of the system's explanations that you found frustrating or that you felt could be improved? What specific changes would you suggest?

## **C.5 Topic 5: Cross-Cultural Understanding and Musical Interpretation**

22. Did learning vocal works in Italian, French, and German change your understanding of these cultures? If so, how?

**[XAI only]** 23. The system sometimes provided cultural context for musical works and performance traditions. Did this cultural information affect how you performed or expressed yourself physically? Can you give a specific example?

24. Do you feel that understanding the cultural background of a vocal work changes how you use your body (gestures, facial expressions, posture) when performing it?

## **C.6 Closing**

25. Is there anything else about your learning experience that we haven't discussed but that you think is important to share?

26. If you could change one thing about the AI-assisted practice system, what would it be and why?

27. Looking back at the entire 16-week experience, what advice would you give to a fellow student about to begin a similar program?
